# Retrospective Evaluation and Framework Development of Bone Anisotropic Material Behavior Compared with Elastic, Elastic-Plastic, and Hyper-Elastic Properties

**DOI:** 10.3390/bioengineering9010009

**Published:** 2021-12-29

**Authors:** Farah Hamandi, James T. Tsatalis, Tarun Goswami

**Affiliations:** 1 Department of Biomedical, Industrial, and Human Factors Engineering, Wright State University, Dayton, OH 45435, USA; tarun.goswami@wright.edu; 2 Department of Radiology, Orthopaedic Surgery, Miami Valley Hospital, Dayton, OH 45409, USA; jttsatalis@premierhealth.com; 3 Department of Orthopedic Surgery, Sports Medicine and Rehabilitation, Wright State University, Dayton, OH 45435, USA

**Keywords:** macroscale, finite element analysis, demography, age, gender, race, anisotropy

## Abstract

The main motivation for studying damage in bone tissue is to better understand how damage develops in the bone tissue and how it progresses. Such knowledge may help in the surgical aspects of joint replacement, fracture fixation or establishing the fracture tolerance of bones to prevent injury. Currently, there are no standards that create a realistic bone model with anisotropic material properties, although several protocols have been suggested. This study seeks to retrospectively evaluate the damage of bone tissue with respect to patient demography including age, gender, race, body mass index (BMI), height, and weight, and their role in causing fracture. Investigators believe that properties derived from CT imaging data to estimate the material properties of bone tissue provides more realistic models. Quantifying and associating damage with in vivo conditions will provide the required information to develop mathematical equations and procedures to predict the premature failure and potentially mitigate problems before they begin. Creating a realistic model for bone tissue can predict the premature failure(s), provide preliminary results before getting the surgery, and optimize the design of orthopaedic implants. A comparison was performed between the proposed model and previous efforts, where they used elastic, hyper- elastic, or elastic-plastic properties. Results showed that there was a significant difference between the anisotropic material properties of bone when compared with unrealistic previous methods. The results showed that the density is 50% higher in male subjects than female subjects. Additionally, the results showed that the density is 47.91% higher in Black subjects than Mixed subjects, 53.27% higher than Caucasian subjects and 57.41% higher than Asian. In general, race should be considered during modeling implants or suggesting therapeutic techniques.

## 1. Introduction

Hip fracture is one of the serious injuries that affects adults. It is estimated that 1.7 million fractures occur every year worldwide, and this is expected to double by 2040 [1,2]. The number of hip and knee joint replacement surgeries has increased significantly in the last four years and expected to reach 3.5 million total knee replacements in 2030. With such statistics, there is a need to understand bone structure more fully and how the damage develops and grows. Modeling bone damage growth may be beneficial to the industry designing implants. Understanding the hierarchical structure of the bone at different levels provides better knowledge about bone tissue mechanical properties and the effect of each level on damage accumulation.

As the number of hip fracture cases occurring worldwide is expected to increase and double by 2040, there is a need to understand how the damage nucleates and grows. The resolution in experimental testing is global or limited by the positioning of strain gages, while the damage at local regions is often least understood. Therefore, more studies of damage development are required at the same time that higher resolution imaging modalities are needed to support construction of 3D bone models. In clinical settings, magnetic resonance imaging (MRI) and computer tomography (CT) use a resolution of 4 mm per slice, which is too global for this type of investigation. As a result, higher resolution imaging methodology might provide more valuable input. Correspondingly, an accurate damage prediction model for bone tissue is needed in order to predict the fracture of the bone or the reliability of a bone-implant structure. Numerous damage models were proposed using the macro and micro structures of the bone [3,4,5,6,7,8,9,10,11,12,13,14,15,16]. However, each model has made an assumption regarding the mechanical properties, loading conditions, or the structure of the bone. These assumptions have not given realistic predictions for the damage accumulation in bone tissue. At the macro-level, the structure of the bone is divided into cortical and cancellous structures. The cortical bone is more dense and easy to be modeled by using CT scans. However, the cancellous bone is harder and needs high resolution images. Cortical and cancellous bones have different structures and material properties. By ignoring either of these bone structures, we would not be able to provide realistic results for bone damage nucleation, propagation, and eventually the complete failure. At the micro-level, it is important to understand the trabeculae orientation, porosity, and interconnectivity. These features are very important to understand the pathology of the bone, which should give a better understanding of whether or not to use an implant and its type in the fracture fixation.

The responses of the bone can be either elastic, plastic, or hyper-elastic. The elastic response of the bone means that when the load is applied, the bone deforms and when the force is removed, the bone returns to its original extent. The plastic response of the bone means that when a specific amount of load is applied, the bone deforms. However, when the force is removed, the bone does not return to its original extent. The hyper-elastic response of the bone is that when the load is applied, the bone deforms, and when the force is removed, the bone returns to its original extent, which is similar to the elastic response. The hyper-elastic strain energy density functions models that were first used were Neo-Hookean and Mooney-Rivilin, and the literature has discussed some models to develop the strain energy density function for the hyper-elastic response.

Additionally, it is well established that there are significant differences between male and female bone shape and structure. According to the American Joint Replacement Registry (AJRR), the knee and hip joint replacement surgeries in females were 61.1% for knee joint and 55.5% for hip joint, for the last four years. Since higher number of females receives these procedures, there is a need to investigate the bone damage development specific to a gender depending upon the imaging data compiled. Age is another significant factor that plays a major role in joint replacement. AJRR showed that 62.3% of hip joint replacement surgeries were in patients with ages between 55–74 years old, and 68.2% of knee joint replacement surgeries were in the same age range (55–74 years old). Such high percentage means that understanding the structural changes in the long bone and the mechanical changes with age would play a significant role in damage development.

The objective of this research is to retrospectively investigate bone mechanics at a macro level and develop a novel standard for bone anisotropic material properties featuring gender, age, race, and bone pathology. This investigation will allow for the using of the imaging modalities to characterize and model damage development in long bones at the macro level. This modeling will provide a full understanding of the mechanical behavior of bone tissue and engineering materials used as implants and the interface between implant and bone as well as how damage grows globally in bones. The proposed research will have a major impact on designing orthopaedic implants and preventing premature failures.

## 2. Materials and Methods

### 2.1. Subjects Preparation

**Figure 1:**
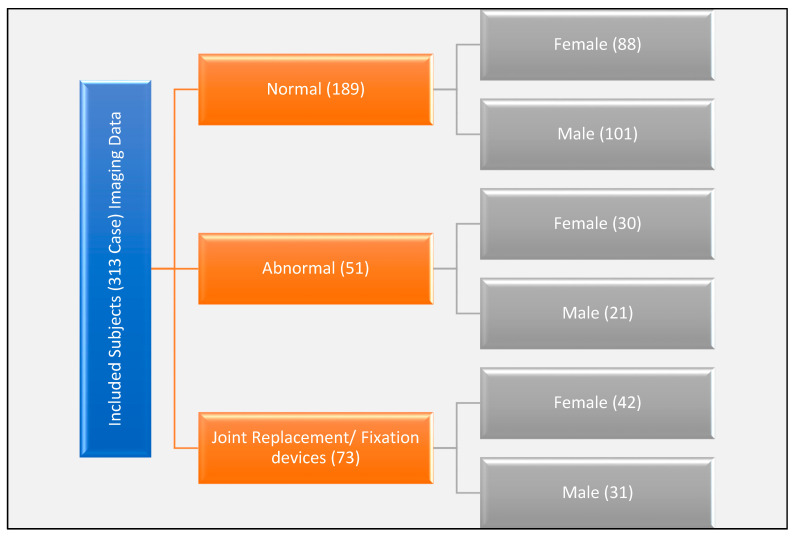
The classification of the 313 subjects included in the study.

In this study, 313 subjects were investigated (Figure 1), and three-dimensional models were developed from CT imaging data. Research was conducted in accordance with the ethics protocol approved by the Health and Research Board (# 06413) at Wright State University, USA. Imaging data collection focused on lower extremity long bones and divided into three major categories, including normal, fractured, and bone with fixation devices. Data collected included demography such as age, gender, race, body mass index (BMI), height, and weight, as well as the clinical indication reported by the radiologist. We classified the selected subjects into Caucasian, Black, Asian, and Mixed, and their ages were from 23–95 years old for female subjects and from 26–92 years old for male subjects.

### 2.2. Material Representation

**Figure 2:**
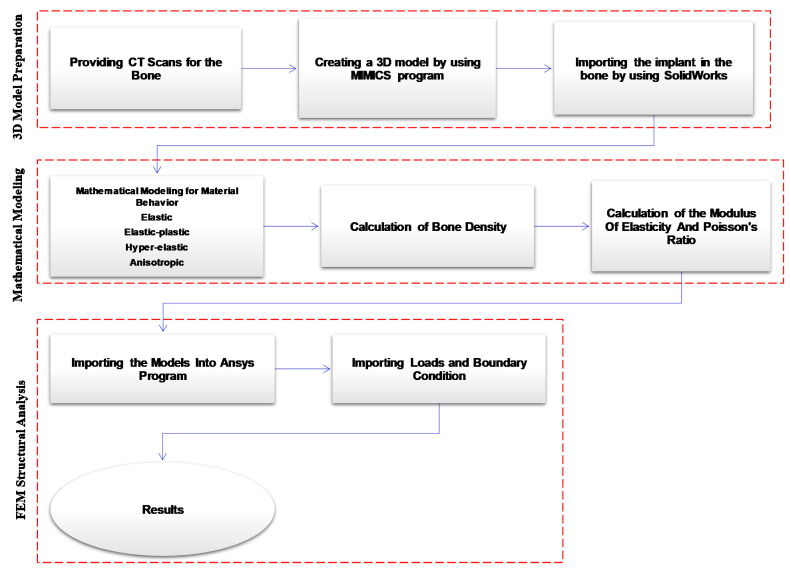
The framework for modeling different material behavior for the bone.

To be able to understand the importance of modeling the bone as an anisotropic structure, it was essential to investigate the difference in the mechanical behavior of the bone with respect to material property representation. Four different material properties were investigated, including elastic, elastic-plastic, and hyper-elastic properties. In addition, Figure 2 shows the framework for modeling different material behavior of the bone.

### 2.3. Finite Element Modeling

Computational modeling for bone tissue offers a deep understanding and provides a considerable amount of information that might be difficult or impossible to discover experimentally. To perform the simulation, the models were created using the MIMICS program. Second, Hounsfield Units (HU) were used to define the material properties. Third, the models and their material properties were imported in the Ansys program to perform the simulation. Fourth, loads and boundary conditions were defined to cope with the daily walking gait cycle. Finally, meshing and simulations were performed. In general, von Mises stresses were investigated for all the 313 subjects and the results were compared with respect to each subject demography.

#### 2.3.1. Creating the Model

**Figure 3:**
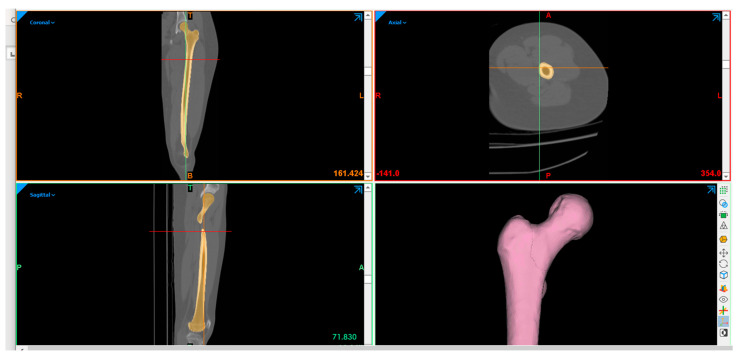
Creating the 3D model of the femoral bone using MIMICS.

To create a three-dimensional model for the bone, CT scans for femoral bone were imported into the MIMICS 13.0 program, as shown in Figure 3. The model was divided into eight segments, which is important for material properties assigning and in applying loads and boundary conditions later. Finally, SolidWorks (Dassault Systèmes SolidWorks Corp., Concord, MA, USA) was used to modify the model.

#### 2.3.2. Material Definition

Four material properties were assigned to the femoral bone model, which are elastic material properties, hyper- elastic material properties, elastic-plastic material properties, and anisotropic material properties. The same femoral bone model was used in this analysis. All of the above material properties parameters were derived from the density of the bone. The same procedure that has been used in the published paper [17] was used to find the modulus of elasticity, Poisson’s ratio, and shear modus from the density of the bone depending on Hounsfield units.

For the elastic material properties, the model was defined as elastic in the Ansys program engineering data, and only the modulus of elasticity and Poisson’s Ratio were needed to define the elastic material. For this model, the density (ρ) of the cortical part was 1.15802 g/cm^3^ and the density of the cancellous part was 0.93508 g/cm^3^. The Young’s modulus was calculated for each part as it equals to $(2314\rho^{1.57}$) for the cortical part and ($1157\rho^{1.78})$ for the trabecular part. Poisson’s Ratio was imported as 0.25 in the program.

For the hyper-elastic material properties, the model was defined as hyperelastic in the Ansys program and the Mooney-Rivlin three parameter model that represents an improvement for the neo-Hookean model was used. Additionally, the same density was used as the elastic model, where it was 1.15802 g/cm^3^ and 0.93508 g/cm^3^ for the cortical and trabecular parts, respectively. The compression test data were taken from our previous experimental work on femur [18] and imported into Ansys, where the program generated the three material constants and the material incompressibility parameter.

For the elastic-plastic material properties, the density, Young’s modulus, and Poisson’s Ratio were imported into Ansys material properties engineering data. For this model, the density of the cortical part used was 1.15802 g/cm^3^ and the density of the trabecular part that was used was 0.93508 g/cm^3^. The Young’s modulus was calculated for each part as it equals to $(2314\rho^{1.57}$) for the cortical part and ($1157\rho^{1.78})$ for the trabecular part. Poisson’s Ratio was imported as 0.25 in the program. To define the plastic range in Ansys, the compressive strength, fracture strain percentage, and hardness needed to be manually imported into the material properties. The ultimate compressive strength was imported as 100 MPa to the cortical bone and 10 MPa to the trabecular bone. The fracture strain % was imported as 3 and 7 to the cortical and trabecular parts, respectively. Additionally, the hardness was imported as 100 into the material properties.

**Figure 4:**
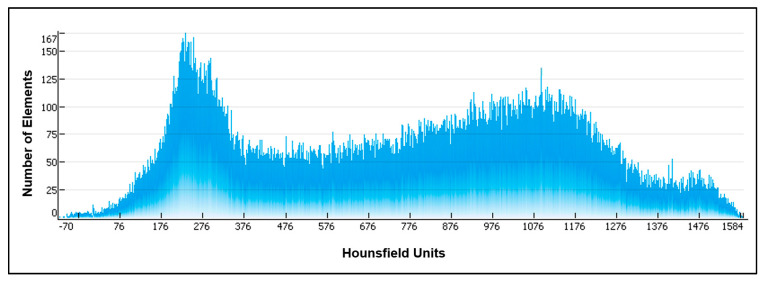
Hounsfield units (HU) distribution across the femoral bone CT images.

For the anisotropic material properties, ten material groups were created for each segment of the bone model. This procedure is very important to provide more realistic approximations for the trabecular bone. The Mimics program was used to find the Hounsfield units across the CT scans of the femoral bone, as shown in Figure 4.

The mathematical relationship between the density and Hounsfield’s units was imported into Mimics program to calculate the density, as follows:

ρ=0.0000464 HU+1

After that, the mathematical relationship between the density and the modulus of elasticity was imported in Mimics. The mathematical relationships between the modulus of elasticity and shear modulus with density that have been imported into Mimics, where different equations were used for the cortical bone segments and the cancellous bone segments. In addition, the table for all the calculated elastic constants is shown in Supplementary Materials A. Also, Table 1 shows the material properties of the cortical and trabecular bone that have been imported into the Ansys program for the elastic, elastic-plastic, and hyper-elastic models.

The Anisotropic relationships between elastic constants and density that were imported into Mimics are as follows [17,19,20,21],

For Cortical Bone Modulus of Elasticity,

(1) $$E_{1}=2314\rho^{1.57}\;$$

(2) $$E_{2}=2314\rho^{1.57}$$

(3) $$E_{3}=2065\rho^{3.09}\;$$

Cortical Bone Shear Modulus

(4) $$G_{12}=\frac{G_{12\;max}\rho^{2}}{\rho_{max}^{2}}.\;$$

(5) $$G_{23}=\frac{G_{23\;max}\rho^{2}}{\rho_{max}^{2}}.\;$$

(6) $$G_{31}=\frac{G_{31\;max}\rho^{2}}{\rho_{max}^{2}}.\;$$

were Poisson Ratio ν_12_ = 0.4, ν_23_ = 0.25, ν_31_ = 0.25.

For Trabecular Bone Modulus of Elasticity,

(7) $$E_{1}=1157\rho^{1.78}$$

(8) $$E_{2}=1157\rho^{1.78}$$

(9) $$E_{3}=1904\rho^{1.64}$$

Trabecular Bone Shear Modulus

(10) $$G_{12}=\frac{G_{12\;max}\rho^{2}}{\rho_{max}^{2}}$$

(11) $$G_{23}=\frac{G_{23\;max}\rho^{2}}{\rho_{max}^{2}}$$

(12) $$G_{31}=\frac{G_{31\;max}\rho^{2}}{\rho_{max}^{2}}$$

where ν_12_ = 0.4, ν_23_ = 0.25, ν_31_ = 0.25, *G*_12 max_ = 5.71 MPa, *G*_23 max_ = 7.11 MPa, and *G*_31 max_ = 6.58 MPa.

#### 2.3.3. Damage Accumulation

The cumulative damage failure of the bone is calculated using the computational simulation in Ansys. The finite element modeling of damage considers that the bone damage equal to (0) when the area is undamaged, while the damage is equal to (1) when the area failed.

#### 2.3.4. Meshing

**Figure 5:**
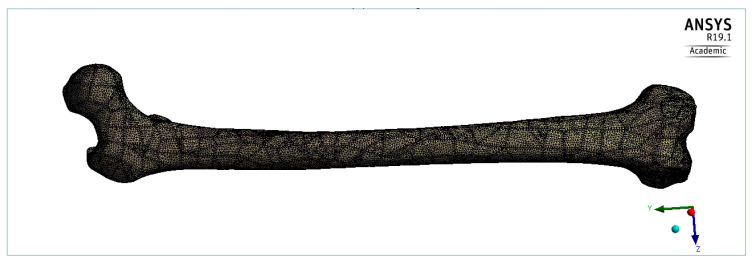
Meshing with tetrahedral elements.

The meshing of the femoral bone model was performed into Ansys Workbench R19.1. A tetrahedral element was used in this analysis with 0.02 mm element size. The number of elements was 400,317 ± 76,922 for the femoral models. Figure 5 shows the meshing of a model of a normal, 41-year-old Caucasian male.

#### 2.3.5. Loads and Boundary Conditions

To mimic the individual realistic loading during walking, the gait cycle loads (taken from HIP98^®^ program) was applied as a time-dependent analysis along the bone longitudinal axis. The load was applied to the femoral head. The femoral condyles were assumed to be fixed inferiorly.

## 3. Results

### 3.1. The Effect of Different Mechanical Properties

**Figure 6:**
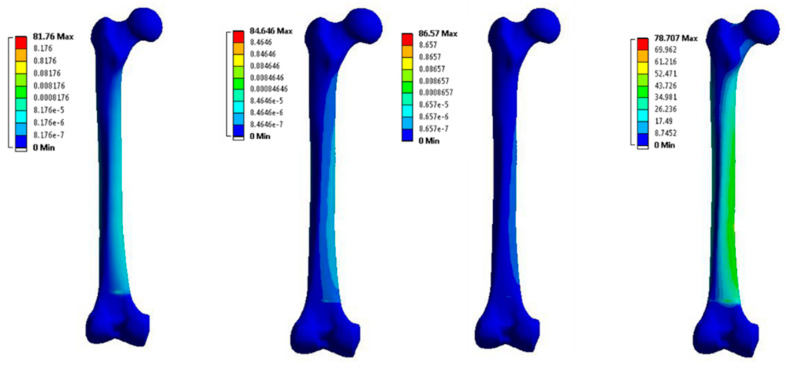
The von-Misses stress (MPa) of four femoral bone models with elastic, elastic-plastic, hyper-elastic, and anisotropic material properties (from **left** to **right**).

**Figure 7:**
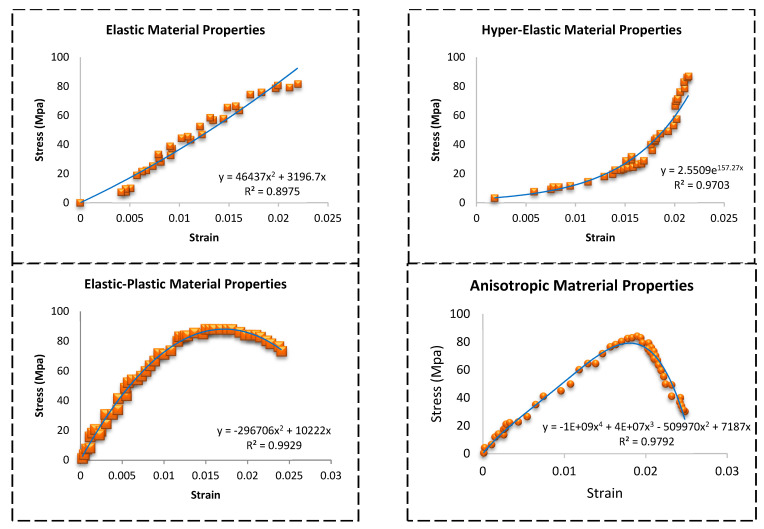
Stress versus strain curves were plotted depending on the finite element analysis results for each model.

**Figure 8:**
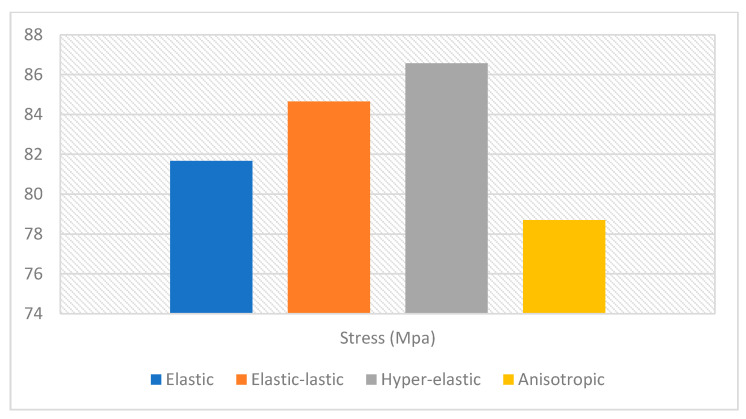
A comparison between the maximum von-Misses stresses for the four models.

The analysis was done by using the Ansys program on the four different models for the same femoral bone but with four different material properties. The von-Misses stresses distribution for the four models are shown in Figure 6. The maximum von-Misses stress for the femoral bone with anisotropic material properties was 78.707 MPa, for the femoral bone with elastic material properties it was 81.67 MPa, for the femoral bone with elastic-plastic material properties it was 84.646 MPa, and finally for the femoral bone with hyper-elastic material properties it was 86.57 MPa. Also, the results presented that the stresses were higher by 1.80% in the model with elastic properties by the comparison with the anisotropic model, the stresses were higher by 3.60% in the model with elastic-plastic properties by the comparison with the anisotropic model, and finally the stresses were higher by 4.70% in the model with hyper-elastic properties by the comparison with the anisotropic model. In addition, stress vs. strain curves were plotted depending on the finite element analysis results for each model, as shown in Figure 7. The regression equations for the four models were also provided for each curve. A comparison between the maximum von-Misses stresses for the four models is shown in Figure 8.

### 3.2. The Effect of Age

**Figure 9:**
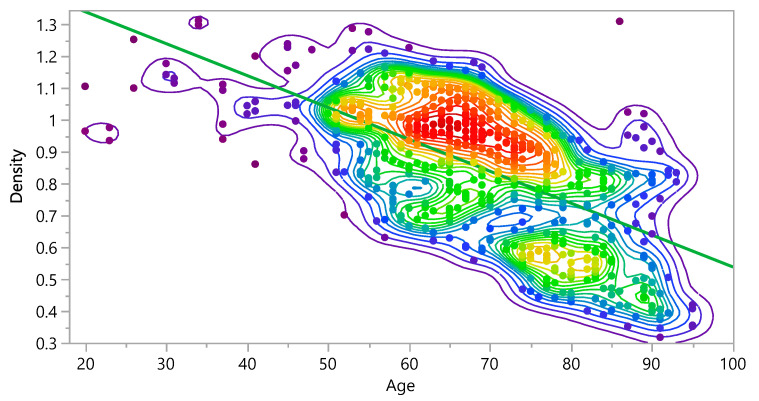
Bivariate Fit of Density by Age.

The impact of age on the density of the bone and eventually on the stress distribution and damage accumulation was investigated. Figure 9 shows that the density decreases as the age increases. Additionally, the results showed that the density is 59.1% higher in young subjects than middle age subjects and 72.2% higher than in older subjects. The bivariate fit shows the relation between density and age, as follows:

Density =1.5404122−0.010025× Age

### 3.3. The Effect of Gender

**Figure 10:**
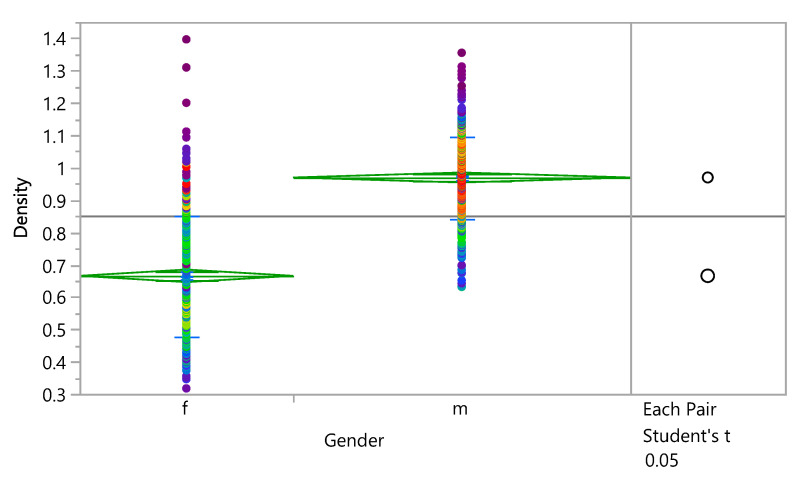
Oneway Analysis of Density by Gender.

**Figure 11:**
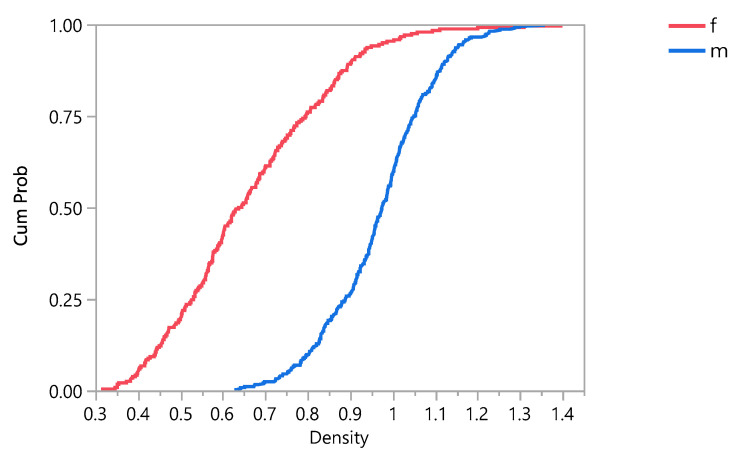
Cumulative damage failure (CFD) plot comparing both genders, where f = female, and m = male.

The impact of gender on the density of the bone and eventually on the stress distribution and damage accumulation was investigated. The results show that the mean density in female subjects was (0.6653613 ± 0.187628 g/cm^3^), and it was (0.9693199 ± 0.1268695 g/cm^3^) in male subjects. Figure 10 shows that the density is higher in males than females with the same ages. Additionally, the results showed that the density is 50% higher in male subjects than female subjects. A Student’s t test connecting letter report showed that there is a significant difference between male and female with 95% confidence interval, as shown in Supplementary Materials B. Cumulative damage failure (CFD) was investigated. Figure 11 shows that the Cumulative damage failure is higher in females than males.

### 3.4. The Effect of Race

**Figure 12:**
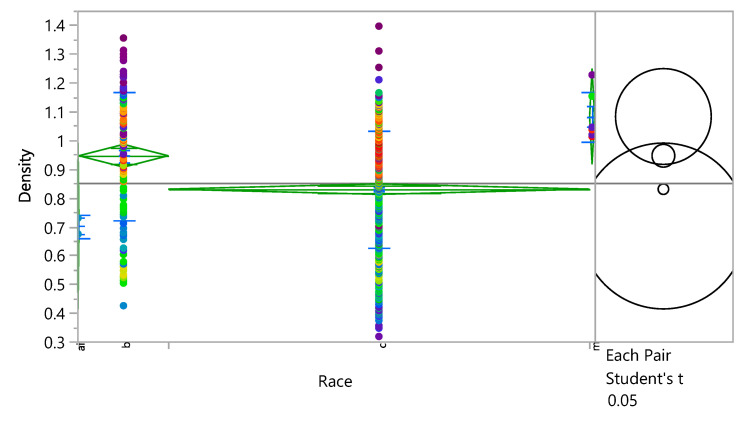
Oneway Analysis of Density by Race.

**Figure 13:**
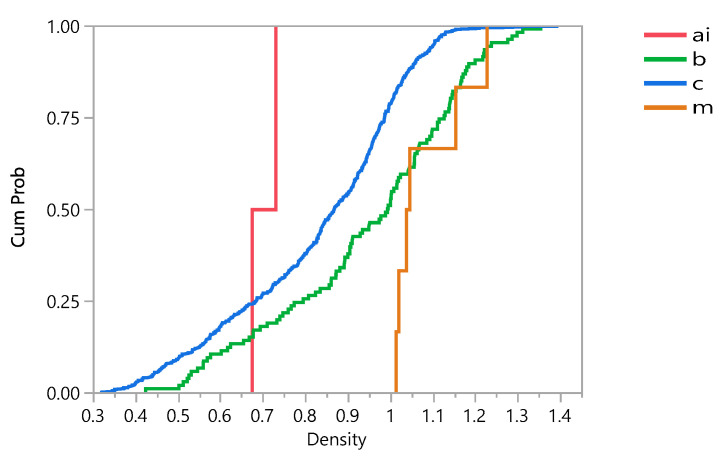
Cumulative damage failure (CFD) plot comparing different races, where ai = Asian, b = Black, c = Caucasian, and m = Mixed.

The impact of race on the density of the bone and eventually on the stress distribution and damage accumulation was investigated. Figure 12 shows that the density is significantly affected by race. Additionally, the results showed that the density is 47.91% higher in Black subjects than Mixed subjects, 53.27% higher than Caucasian subjects and 57.41% higher than Asian. A Student’s t test shows that there are significant differences among different races (with 95% confidence interval). Figure 13 shows that cumulative damage failure is higher in Caucasian cases. In general, gender, race, and age showed significant effects on the damage distribution.

### 3.5. The Effect of BMI, Weight, and Height

**Figure 14:**
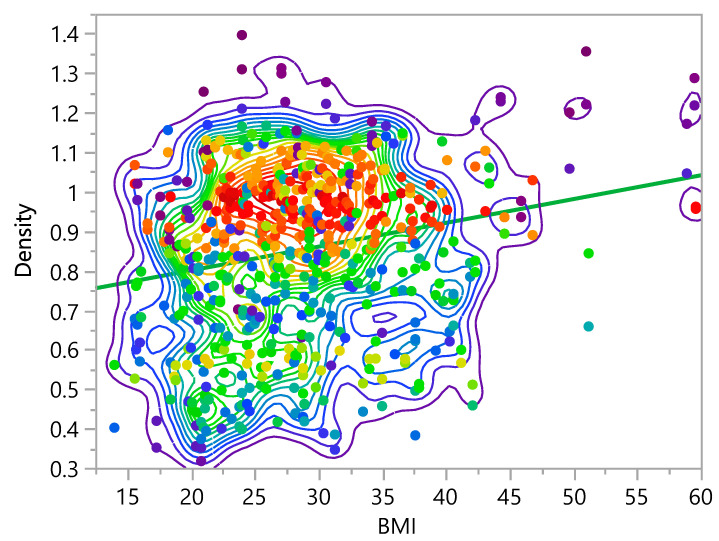
Bivariate Fit of Density by BMI.

**Figure 15:**
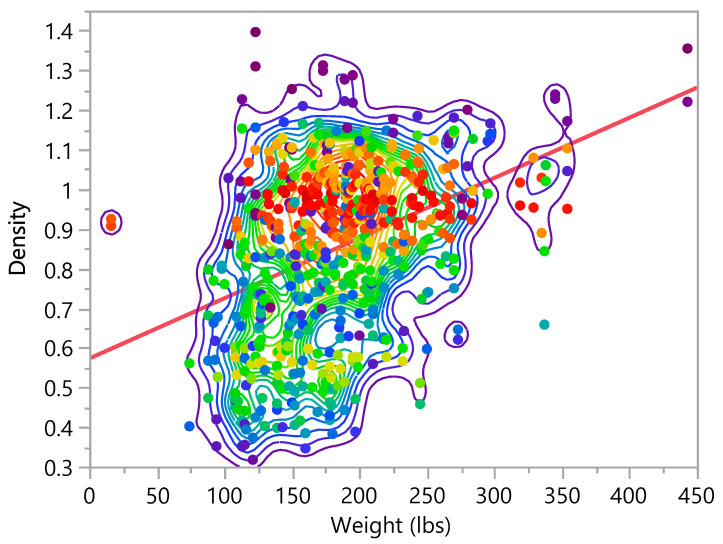
Bivariate Fit of Density by Weight (lbs).

**Figure 16:**
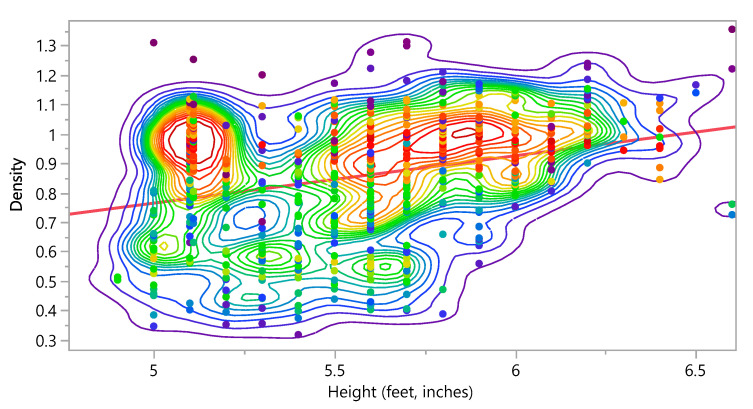
Bivariate Fit of Density by Height (feet, inches).

The statistical analysis showed that the density increases as the BMI, weight, and height increase, as shown in Figure 14, Figure 15 and Figure 16. The bivariate fit shows the relation between density and age, as follows:

Density =0.6791666+0.0060304× BMI

Density =0.5729131+0.0015183× Weight

Density =−0.040764+0.1609694× Height

### 3.6. The von Mises Stress

**Figure 17:**
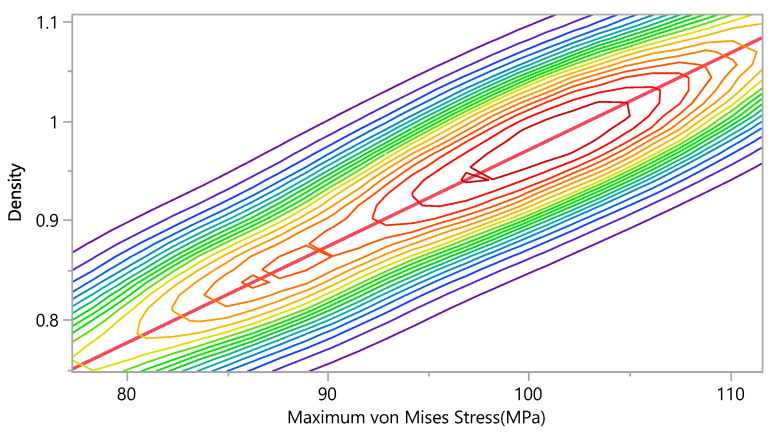
Bivariate Fit of Density by Maximum von Mises Stress (MPa).

The results of the finite element simulations were investigated and the effect of the anisotropic mechanical properties on the stress distribution. The maximum von Mises stresses were compared with the density. Figure 17 shows that the maximum von Mises stresses increases as the density increases. The bivariate fit shows the relation between maximum von Mises stresses and density, as follows:

$$\mathrm{Density}=4.352\times 10^{-11}+\left(\;0.0097057\times \mathrm{Maximum}\;\mathrm{von}\;\mathrm{Mises}\;\mathrm{Stress}\right)$$

## 4. Discussion

This study involves retrospective evaluation and framework development of bone anisotropic material behavior compared with elastic, elastic-plastic, and hyper-elastic properties. This chapter seeks to retrospectively evaluate the damage of bone tissue of 313 subjects with respect to patient demography including age, gender, race, body mass index (BMI), height, and weight, and their role in causing fracture. Currently, there are no standards that create a realistic bone model with anisotropic material properties, although several protocols have been suggested. This study seeks to retrospectively evaluate the damage of bone tissue with respect to patient demography including age, gender, race, body mass index (BMI), height, and weight, and their role in causing fracture. Investigators believe that properties derived from CT imaging data to estimate the material properties of bone tissue provides more realistic models. Quantifying and associating damage with in vivo conditions will provide the required information to develop mathematical equations and procedures to predict the premature failure and potentially mitigate problems before they begin. Creating a realistic model for bone tissue can predict the premature failure(s), provide preliminary results before getting the surgery, and optimize the design of orthopaedic implants. A comparison was performed between the proposed model and previous efforts, where they used elastic, hyper- elastic, or elastic-plastic properties. Results showed that there was a significant difference between the anisotropic material properties of bone when compared with unrealistic previous methods. The results showed that the density is 50% higher in male subjects than female subjects. Additionally, the results showed that the density is 47.91% higher in Black subjects than Mixed subjects, 53.27% higher than Caucasian subjects and 57.41% higher than Asian. In general, race should be considered during modeling implants or suggesting therapeutic techniques.

## 5. Conclusions

Quantifying and associating damage with in vivo conditions provides the required information to develop mathematical equations and procedures to predict premature failure and potentially mitigate problems before they begin. Creating a realistic model for bone tissue can predict premature failure(s), provide preliminary results before performing the surgery, and optimize the design of orthopaedic implants. The proposed method can be used as a standard that creates a realistic bone model with anisotropic material properties. Additionally, the proposed method can be used in customized diagnostic techniques or in navigation systems to provide accurate predictions before performing surgery. Additionally, gender has a significant effect on the density of the bone. More precautions should be taken into consideration for older females. Furthermore, race should be considered during modeling implants or suggesting therapeutic techniques. Caucasian subjects have the least density of any other race with the same age and gender. In general, Age is a significant factor and has an essential effect on the mechanical properties of the bone. The density and maximum von Mises stress decreases drastically in the elderly, which means using the same fixation devices and implants as on younger cases is not reasonable. Different therapeutic techniques should be considered for older patients.

## Figures and Tables

**Table 1:** Material properties of the cortical and trabecular bone.

Material Properties	Cortical Bone	Trabecular Bone
Young’s modulus (GPa)	$E=2314\rho^{1.57}$	$E=1157\rho^{1.78}$
Poisson’s Ratio	0.25	0.25
Ultimate Tensile strength (MPa)	100	10
Ultimate Compressive strength (MPa)	100	10
Fracture strain %	1–3	5–7
Toughness (MPa.m^1/2^)	2	-
Hardness (Vickers)	50–100	-
Shear modulus (GPa)	4959	-
Ultimate Tensile strain	0.0083	-
Ultimate Compressive strain	0.0083	-
Ultimate Shear strain	0.0202	-

## Data Availability

Not applicable.

## Supplementary Materials

The following supporting information can be downloaded at: https://www.mdpi.com/article/10.3390/bioengineering9010009/s1, Supplementary materials A: The elastic constants, Supplementary materials B: The Statistical Analysis.
