# Supplementary material for: Retrospective Evaluation and Framework Development of Bone Anisotropic Material Behavior Compared with Elastic, Elastic-Plastic, and Hyper-Elastic Properties
Source: Bioengineering (Basel). 2021 Dec 29;9(1):9. doi: 10.3390/bioengineering9010009 (PMC8773132; doi:10.3390/bioengineering9010009)
Supplement: Supplementary file 1 [file bioengineering-09-00009-s001.zip › bioengineering-1420470-supplementary.pdf]

Supplementary materials A

The elastic constants

No.	Race	Age	Gender	Weight (lbs)	Height (feet, inches)	BMI	Cortical	Trabeculur	Side	E1 (GPa)	E2 (GPa)	E3 (GPa)	v12	v23	v31	G12	G23	G31
**1**	c	23	f	276	5.5	45.9	1.15802	0.93508	Right	1.027	1.027	1.706	0.400	0.250	0.250	0.046	0.058	0.053
**2**	c	23	f	276	5.5	45.9	1.11037	0.97599	Left	1.108	1.108	1.830	0.400	0.250	0.250	0.051	0.063	0.058
**5**	b	37	f	176	5.6	28.4	1.275308	1.111324	Right	1.396	1.396	2.264	0.400	0.250	0.250	0.066	0.082	0.076
**6**	b	37	f	176	5.6	28.4	1.227658	1.093279	Left	1.356	1.356	2.204	0.400	0.250	0.250	0.063	0.079	0.073
**7**	m	40	f	214	5.8	32.5	1.200805	1.044722	Right	1.251	1.251	2.046	0.400	0.250	0.250	0.058	0.072	0.067
**8**	m	40	f	214	5.8	32.5	1.153155	1.018775	Left	1.196	1.196	1.963	0.400	0.250	0.250	0.055	0.069	0.064
**9**	b	41	f	280	5.3	49.7	1.286914	1.057654	Right	1.278	1.278	2.087	0.400	0.250	0.250	0.059	0.074	0.068
**10**	b	41	f	280	5.3	49.7	1.334564	1.200184	Left	1.601	1.601	2.568	0.400	0.250	0.250	0.076	0.095	0.088
**11**	c	41	f	103	5.2	18.9	1.114696	0.86099	Right	0.886	0.886	1.490	0.400	0.250	0.250	0.039	0.049	0.045
**12**	c	41	f	103	5.2	18.9	1.162346	1.027966	Left	1.215	1.215	1.992	0.400	0.250	0.250	0.056	0.070	0.065
**13**	c	47	f	138	6.1	18.3	1.084958	0.877674	Right	0.917	0.917	1.537	0.400	0.250	0.250	0.041	0.051	0.047
**14**	c	47	f	138	6.1	18.3	1.037308	0.902928	Left	0.965	0.965	1.610	0.400	0.250	0.250	0.043	0.054	0.050
**15**	c	51	f	152	5.7	24.2	1.054411	0.835433	Right	0.840	0.840	1.418	0.400	0.250	0.250	0.037	0.046	0.043
**16**	c	51	f	152	5.7	24.2	1.102061	0.967681	Left	1.091	1.091	1.804	0.400	0.250	0.250	0.050	0.062	0.057
**17**	c	54	f	141	5.8	21.9	1.019796	0.873569	Right	0.910	0.910	1.525	0.400	0.250	0.250	0.041	0.050	0.047
**18**	c	54	f	130	5.5	21.6	1.009598	0.827355	Right	0.826	0.826	1.395	0.400	0.250	0.250	0.036	0.045	0.042
**19**	c	54	f	141	5.8	21.9	0.972146	0.837766	Left	0.844	0.844	1.424	0.400	0.250	0.250	0.037	0.046	0.043
**20**	c	54	f	130	5.5	21.6	1.057248	0.922868	Left	1.003	1.003	1.669	0.400	0.250	0.250	0.045	0.056	0.052
**21**	c	55	f	173	5.1	32.7	0.9994	0.757805	Right	0.706	0.706	1.208	0.400	0.250	0.250	0.030	0.038	0.035
**22**	c	55	f	173	5.1	32.7	0.95175	0.81737	Left	0.808	0.808	1.368	0.400	0.250	0.250	0.035	0.044	0.041
**23**	c	56	f	229	5.2	41.9	1.001169	0.784242	Right	0.751	0.751	1.278	0.400	0.250	0.250	0.033	0.041	0.038
**24**	c	56	f	157	5.6	25.4	0.981146	0.682268	Right	0.586	0.586	1.017	0.400	0.250	0.250	0.025	0.031	0.028

**25**	c	56	f	223	5.10.	32.1	1.011181	0.841812	Right	0.852	0.852	1.436	0.400	0.250	0.250	0.038	0.047	0.043
**26**	c	56	f	229	5.2	41.9	0.953519	0.81914	Left	0.811	0.811	1.373	0.400	0.250	0.250	0.036	0.044	0.041
**27**	c	56	f	157	5.6	25.4	1.028796	0.894416	Left	0.949	0.949	1.586	0.400	0.250	0.250	0.042	0.053	0.049
**28**	c	56	f	223	5.10.	32.1	1.058831	0.924451	Left	1.006	1.006	1.674	0.400	0.250	0.250	0.045	0.057	0.052
**29**	b	58	f	251	5.6	40.6	0.960014	0.740362	Right	0.678	0.678	1.163	0.400	0.250	0.250	0.029	0.036	0.034
**30**	b	58	f	251	5.6	40.6	1.007664	0.873285	Left	0.909	0.909	1.525	0.400	0.250	0.250	0.040	0.050	0.047
**31**	c	58	f	218	5.7	34.1	0.975886	0.75175	Right	0.696	0.696	1.192	0.400	0.250	0.250	0.030	0.037	0.035
**32**	c	58	f	156	5.1	29.6	0.956368	0.717993	Right	0.642	0.642	1.106	0.400	0.250	0.250	0.027	0.034	0.032
**33**	c	58	f	218	5.7	34.1	0.928236	0.793856	Left	0.767	0.767	1.304	0.400	0.250	0.250	0.033	0.042	0.039
**34**	c	58	f	156	5.1	29.6	0.908718	0.774338	Left	0.734	0.734	1.252	0.400	0.250	0.250	0.032	0.040	0.037
**35**	b	59	f	160	5.6	25.9	1.094391	0.861632	Right	0.888	0.888	1.491	0.400	0.250	0.250	0.039	0.049	0.045
**36**	b	59	f	160	5.6	25.9	1.046741	0.912361	Left	0.983	0.983	1.638	0.400	0.250	0.250	0.044	0.055	0.051
**37**	b	60	f	259	5.4	44.6	1.116726	0.893158	Right	0.946	0.946	1.582	0.400	0.250	0.250	0.042	0.053	0.049
**38**	b	60	f	259	5.4	44.6	1.069076	0.934696	Left	1.026	1.026	1.704	0.400	0.250	0.250	0.046	0.058	0.053
**39**	c	60	f	246	5.6	39.8	0.932479	0.722467	Right	0.649	0.649	1.117	0.400	0.250	0.250	0.028	0.035	0.032
**40**	c	60	f	114	5.4	19.6	0.941994	0.766445	Right	0.721	0.721	1.231	0.400	0.250	0.250	0.031	0.039	0.036
**41**	c	60	f	246	5.6	39.8	0.980129	0.84575	Left	0.859	0.859	1.447	0.400	0.250	0.250	0.038	0.047	0.044
**42**	c	60	f	114	5.4	19.6	0.989644	0.855265	Left	0.876	0.876	1.473	0.400	0.250	0.250	0.039	0.048	0.045
**43**	c	61	f	115	5.5	19.1	0.95151	0.686932	Right	0.593	0.593	1.029	0.400	0.250	0.250	0.025	0.031	0.029
**44**	c	61	f	115	5.5	19.1	0.99916	0.86478	Left	0.893	0.893	1.500	0.400	0.250	0.250	0.040	0.049	0.046
**45**	b	62	f	181	5.9	26.8	1.089424	0.807417	Right	0.791	0.791	1.341	0.400	0.250	0.250	0.035	0.043	0.040
**46**	b	62	f	181	5.9	26.8	1.137074	1.002695	Left	1.163	1.163	1.912	0.400	0.250	0.250	0.053	0.066	0.062
**47**	c	62	f	207	5	40.6	0.911407	0.660264	Right	0.553	0.553	0.964	0.400	0.250	0.250	0.023	0.029	0.027
**48**	c	62	f	207	5	40.6	0.920707	0.724478	Right	0.652	0.652	1.122	0.400	0.250	0.250	0.028	0.035	0.032
**49**	c	62	f	170	5.5	29.3	0.943958	0.699563	Right	0.613	0.613	1.060	0.400	0.250	0.250	0.026	0.032	0.030
**50**	c	62	f	207	5	40.6	0.863757	0.729378	Left	0.660	0.660	1.135	0.400	0.250	0.250	0.028	0.035	0.033
**51**	c	62	f	207	5	40.6	0.873057	0.738678	Left	0.675	0.675	1.159	0.400	0.250	0.250	0.029	0.036	0.033
**52**	c	62	f	170	5.5	29.3	0.991608	0.857228	Left	0.880	0.880	1.479	0.400	0.250	0.250	0.039	0.049	0.045

**53**	c	63	f	203	5.5	33.8	0.939308	0.71419	Right	0.636	0.636	1.096	0.400	0.250	0.250	0.027	0.034	0.031
**54**	c	63	f	203	5.5	33.8	0.986958	0.852578	Left	0.871	0.871	1.466	0.400	0.250	0.250	0.039	0.048	0.044
**55**	b	65	f	140	5.7	22	1.036671	0.893368	Right	0.947	0.947	1.583	0.400	0.250	0.250	0.042	0.053	0.049
**56**	b	65	f	140	5.7	22	1.084321	0.949941	Left	1.056	1.056	1.750	0.400	0.250	0.250	0.048	0.060	0.055
**57**	c	65	f	88	5.2	16.1	0.883897	0.681404	Right	0.585	0.585	1.015	0.400	0.250	0.250	0.025	0.031	0.028
**58**	c	65	f	129	5.4	22.3	0.90413	0.719507	Right	0.644	0.644	1.110	0.400	0.250	0.250	0.027	0.034	0.032
**59**	c	65	f	192	5.5	32.1	0.866219	0.629008	Right	0.507	0.507	0.890	0.400	0.250	0.250	0.021	0.026	0.024
**60**	c	65	f	133	5.6	21.5	0.892736	0.711278	Right	0.631	0.631	1.089	0.400	0.250	0.250	0.027	0.033	0.031
**61**	c	65	f	88	5.2	16.1	0.931547	0.797167	Left	0.773	0.773	1.313	0.400	0.250	0.250	0.034	0.042	0.039
**62**	c	65	f	129	5.4	22.3	0.85648	0.7221	Left	0.648	0.648	1.116	0.400	0.250	0.250	0.028	0.034	0.032
**63**	c	65	f	192	5.5	32.1	0.913869	0.779489	Left	0.743	0.743	1.265	0.400	0.250	0.250	0.032	0.040	0.037
**64**	c	65	f	133	5.6	21.5	0.845086	0.710706	Left	0.630	0.630	1.088	0.400	0.250	0.250	0.027	0.033	0.031
**65**	b	66	f	132	5.4	22.8	1.015937	0.774412	Right	0.734	0.734	1.252	0.400	0.250	0.250	0.032	0.040	0.037
**66**	b	66	f	132	5.4	22.8	1.063587	0.929208	Left	1.015	1.015	1.688	0.400	0.250	0.250	0.046	0.057	0.053
**67**	c	66	f	130	5.4	22.3	0.875058	0.723485	Right	0.650	0.650	1.120	0.400	0.250	0.250	0.028	0.035	0.032
**68**	c	66	f	130	5.4	22.3	0.922708	0.788328	Left	0.758	0.758	1.289	0.400	0.250	0.250	0.033	0.041	0.038
**69**	c	67	f	92	5.4	15.8	0.855833	0.662872	Right	0.557	0.557	0.970	0.400	0.250	0.250	0.023	0.029	0.027
**70**	c	67	f	121	5.6	20	0.838716	0.604344	Right	0.472	0.472	0.834	0.400	0.250	0.250	0.019	0.024	0.022
**71**	c	67	f	100	5.7	15.7	0.847275	0.599777	Right	0.466	0.466	0.823	0.400	0.250	0.250	0.019	0.024	0.022
**72**	c	67	f	92	5.4	15.8	0.903483	0.769103	Left	0.725	0.725	1.238	0.400	0.250	0.250	0.031	0.039	0.036
**73**	c	67	f	121	5.6	20	0.886366	0.751987	Left	0.697	0.697	1.193	0.400	0.250	0.250	0.030	0.037	0.035
**74**	c	67	f	100	5.7	15.7	0.894925	0.760545	Left	0.711	0.711	1.215	0.400	0.250	0.250	0.031	0.038	0.035
**75**	b	69	f	209	5.1	39.5	0.997418	0.766963	Right	0.721	0.721	1.232	0.400	0.250	0.250	0.031	0.039	0.036
**76**	b	69	f	270	5.7	42.3	0.97747	0.824622	Right	0.821	0.821	1.388	0.400	0.250	0.250	0.036	0.045	0.042
**77**	b	69	f	209	5.1	39.5	1.045068	0.910688	Left	0.980	0.980	1.633	0.400	0.250	0.250	0.044	0.055	0.051
**78**	b	69	f	270	5.7	42.3	0.92982	0.79544	Left	0.770	0.770	1.308	0.400	0.250	0.250	0.034	0.042	0.039
**79**	c	69	f	250	5.7	39.2	0.824416	0.596789	Right	0.462	0.462	0.817	0.400	0.250	0.250	0.019	0.024	0.022

**80**	c	69	f	178	4.11	36	0.83266	0.587802	Right	0.449	0.449	0.797	0.400	0.250	0.250	0.018	0.023	0.021
**81**	c	69	f	250	5.7	39.2	0.872066	0.737686	Left	0.673	0.673	1.156	0.400	0.250	0.250	0.029	0.036	0.033
**82**	c	69	f	178	4.11	36	0.88031	0.745931	Left	0.687	0.687	1.177	0.400	0.250	0.250	0.030	0.037	0.034
**83**	b	70	f	204	5.2	37.3	0.987444	0.747699	Right	0.690	0.690	1.182	0.400	0.250	0.250	0.030	0.037	0.034
**84**	b	70	f	204	5.2	37.3	1.035094	0.900714	Left	0.961	0.961	1.604	0.400	0.250	0.250	0.043	0.054	0.050
**85**	c	70	f	218	5.4	37.6	0.807928	0.621432	Right	0.496	0.496	0.873	0.400	0.250	0.250	0.021	0.026	0.024
**86**	c	70	f	158	5	30.9	0.816172	0.658729	Right	0.550	0.550	0.960	0.400	0.250	0.250	0.023	0.029	0.027
**87**	c	70	f	218	5.4	37.6	0.760278	0.625898	Left	0.502	0.502	0.883	0.400	0.250	0.250	0.021	0.026	0.024
**88**	c	70	f	158	5	30.9	0.863822	0.729442	Left	0.660	0.660	1.135	0.400	0.250	0.250	0.028	0.035	0.033
**89**	b	71	f	128	5.11	17.9	0.959232	0.710729	Right	0.630	0.630	1.088	0.400	0.250	0.250	0.027	0.033	0.031
**90**	b	71	f	128	5.11	17.9	1.006882	0.872503	Left	0.908	0.908	1.522	0.400	0.250	0.250	0.040	0.050	0.047
**91**	c	73	f	112	5	21.9	0.787213	0.654441	Right	0.544	0.544	0.950	0.400	0.250	0.250	0.023	0.028	0.026
**92**	c	73	f	112	5	21.9	0.739563	0.605183	Left	0.473	0.473	0.836	0.400	0.250	0.250	0.019	0.024	0.022
**93**	c	74	f	195	5.1	36.8	0.757866	0.565863	Right	0.420	0.420	0.748	0.400	0.250	0.250	0.017	0.021	0.020
**94**	c	74	f	199	5.4	34	0.757866	0.550532	Right	0.400	0.400	0.715	0.400	0.250	0.250	0.016	0.020	0.019
**95**	c	74	f	151	5.3	26.8	0.765599	0.533995	Right	0.379	0.379	0.680	0.400	0.250	0.250	0.015	0.019	0.017
**96**	c	74	f	115	5	22.6	0.781066	0.54073	Right	0.387	0.387	0.695	0.400	0.250	0.250	0.016	0.019	0.018
**97**	c	74	f	195	5.1	36.8	0.710216	0.575836	Left	0.433	0.433	0.770	0.400	0.250	0.250	0.018	0.022	0.020
**98**	c	74	f	199	5.4	34	0.710216	0.575836	Left	0.433	0.433	0.770	0.400	0.250	0.250	0.018	0.022	0.020
**99**	c	74	f	151	5.3	26.8	0.813249	0.678869	Left	0.581	0.581	1.009	0.400	0.250	0.250	0.024	0.030	0.028
**100**	c	74	f	115	5	22.6	0.733416	0.599036	Left	0.465	0.465	0.822	0.400	0.250	0.250	0.019	0.024	0.022
**101**	c	75	f	148	5.5	24.6	0.74285	0.502514	Right	0.340	0.340	0.616	0.400	0.250	0.250	0.013	0.017	0.015
**102**	c	75	f	148	5.5	24.6	0.7905	0.65612	Left	0.546	0.546	0.954	0.400	0.250	0.250	0.023	0.028	0.026
**103**	c	77	f	162	5	31.67	0.731137	0.596757	Right	0.462	0.462	0.817	0.400	0.250	0.250	0.019	0.024	0.022
**104**	c	77	f	108	5	21.1	0.75801	0.485314	Right	0.319	0.319	0.582	0.400	0.250	0.250	0.013	0.016	0.014
**105**	c	77	f	165	5.7	25.9	0.738449	0.50012	Right	0.337	0.337	0.611	0.400	0.250	0.250	0.013	0.017	0.015
**106**	c	77	f	162	5	31.67	0.778787	0.644407	Left	0.529	0.529	0.926	0.400	0.250	0.250	0.022	0.027	0.025
**107**	c	77	f	108	5	21.1	0.71036	0.575981	Left	0.433	0.433	0.770	0.400	0.250	0.250	0.018	0.022	0.020

**108**	c	77	f	165	5.7	25.9	0.690799	0.556419	Left	0.408	0.408	0.728	0.400	0.250	0.250	0.016	0.020	0.019
**109**	c	78	f	173	5.5	28.8	0.716514	0.488364	Right	0.323	0.323	0.588	0.400	0.250	0.250	0.013	0.016	0.015
**110**	c	78	f	147	5	28.7	0.723826	0.60363	Right	0.471	0.471	0.832	0.400	0.250	0.250	0.019	0.024	0.022
**111**	c	78	f	127	5.3	22.7	0.706946	0.44099	Right	0.269	0.269	0.497	0.400	0.250	0.250	0.010	0.013	0.012
**112**	c	78	f	173	5.5	28.8	0.668864	0.534485	Left	0.379	0.379	0.682	0.400	0.250	0.250	0.015	0.019	0.017
**113**	c	78	f	147	5	28.7	0.771476	0.637096	Left	0.519	0.519	0.909	0.400	0.250	0.250	0.022	0.027	0.025
**114**	c	78	f	127	5.3	22.7	0.754596	0.620216	Left	0.494	0.494	0.870	0.400	0.250	0.250	0.020	0.025	0.024
**115**	c	79	f	245	5.4	42.1	0.692709	0.457893	Right	0.288	0.288	0.529	0.400	0.250	0.250	0.011	0.014	0.013
**116**	c	79	f	220	5.7	34.5	0.706946	0.576803	Right	0.434	0.434	0.772	0.400	0.250	0.250	0.018	0.022	0.020
**117**	c	79	f	245	5.4	42.1	0.645059	0.510679	Left	0.350	0.350	0.632	0.400	0.250	0.250	0.014	0.017	0.016
**118**	c	79	f	220	5.7	34.5	0.754596	0.620216	Left	0.494	0.494	0.870	0.400	0.250	0.250	0.020	0.025	0.024
**119**	c	80	f	179	5	35	0.706846	0.456319	Right	0.286	0.286	0.526	0.400	0.250	0.250	0.011	0.014	0.013
**120**	c	80	f	179	5	35	0.659196	0.524816	Left	0.367	0.367	0.661	0.400	0.250	0.250	0.015	0.018	0.017
**121**	b	82	f	125	5.7	19.7	0.819924	0.560414	Right	0.413	0.413	0.737	0.400	0.250	0.250	0.017	0.021	0.019
**122**	b	82	f	109	5.4	18.7	0.764808	0.531199	Right	0.375	0.375	0.675	0.400	0.250	0.250	0.015	0.019	0.017
**123**	b	82	f	125	5.7	19.7	0.867574	0.733194	Left	0.666	0.666	1.145	0.400	0.250	0.250	0.029	0.036	0.033
**124**	b	82	f	109	5.4	18.7	0.812458	0.678078	Left	0.579	0.579	1.007	0.400	0.250	0.250	0.024	0.030	0.028
**125**	c	82	f	232	5.3	41.2	0.68555	0.565981	Right	0.420	0.420	0.749	0.400	0.250	0.250	0.017	0.021	0.020
**126**	c	82	f	143	5.7	22.4	0.678695	0.399434	Right	0.226	0.226	0.423	0.400	0.250	0.250	0.008	0.011	0.010
**127**	c	82	f	232	5.3	41.2	0.7332	0.598821	Left	0.464	0.464	0.821	0.400	0.250	0.250	0.019	0.024	0.022
**128**	c	82	f	143	5.7	22.4	0.631045	0.496665	Left	0.333	0.333	0.604	0.400	0.250	0.250	0.013	0.016	0.015
**129**	b	83	f	186	5.6	30.2	0.780416	0.546478	Right	0.395	0.395	0.707	0.400	0.250	0.250	0.016	0.020	0.018
**130**	b	83	f	120	5.7	18.8	0.741464	0.521811	Right	0.364	0.364	0.655	0.400	0.250	0.250	0.014	0.018	0.017
**131**	b	83	f	186	5.6	30.2	0.828066	0.693687	Left	0.603	0.603	1.045	0.400	0.250	0.250	0.026	0.032	0.029
**132**	b	83	f	120	5.7	18.8	0.693814	0.559434	Left	0.411	0.411	0.734	0.400	0.250	0.250	0.017	0.021	0.019
**133**	c	83	f	140	5.2	25.6	0.772612	0.531017	Right	0.375	0.375	0.674	0.400	0.250	0.250	0.015	0.019	0.017
**134**	c	83	f	169	5.5	28.2	0.671839	0.437928	Right	0.266	0.266	0.492	0.400	0.250	0.250	0.010	0.013	0.012
**135**	c	83	f	140	5.2	25.6	0.724962	0.590582	Left	0.453	0.453	0.803	0.400	0.250	0.250	0.019	0.023	0.021

**136**	c	83	f	169	5.5	28.2	0.719489	0.58511	Left	0.446	0.446	0.791	0.400	0.250	0.250	0.018	0.023	0.021
**137**	b	84	f	123	5.1	23.2	0.663859	0.424143	Right	0.251	0.251	0.466	0.400	0.250	0.250	0.010	0.012	0.011
**138**	b	84	f	126	5.3	22.3	0.741464	0.60255	Right	0.470	0.470	0.830	0.400	0.250	0.250	0.019	0.024	0.022
**139**	b	84	f	123	5.1	23.2	0.711509	0.57713	Left	0.435	0.435	0.773	0.400	0.250	0.250	0.018	0.022	0.020
**140**	b	84	f	126	5.3	22.3	0.789114	0.654734	Left	0.544	0.544	0.951	0.400	0.250	0.250	0.023	0.028	0.026
**141**	c	85	f	108	5	21.1	0.653894	0.450504	Right	0.280	0.280	0.515	0.400	0.250	0.250	0.011	0.013	0.012
**142**	c	85	f	139	5.2	25.5	0.640816	0.510037	Right	0.349	0.349	0.631	0.400	0.250	0.250	0.014	0.017	0.016
**143**	c	85	f	74	5.1	14	0.647355	0.401627	Right	0.228	0.228	0.427	0.400	0.250	0.250	0.009	0.011	0.010
**144**	c	85	f	108	5	21.1	0.606244	0.471864	Left	0.304	0.304	0.556	0.400	0.250	0.250	0.012	0.015	0.014
**145**	c	85	f	139	5.2	25.5	0.688466	0.554086	Left	0.404	0.404	0.723	0.400	0.250	0.250	0.016	0.020	0.019
**146**	c	85	f	74	5.1	14	0.695005	0.560625	Left	0.413	0.413	0.737	0.400	0.250	0.250	0.017	0.021	0.019
**147**	c	86	f	123	5	24	1.396307	1.396307	Right	2.096	2.096	3.292	0.400	0.250	0.250	0.104	0.129	0.119
**148**	c	86	f	123	5	24	1.443957	1.309577	Left	1.870	1.870	2.963	0.400	0.250	0.250	0.091	0.113	0.105
**149**	b	89	f	93	5.4	16	0.799176	0.569273	Right	0.424	0.424	0.756	0.400	0.250	0.250	0.017	0.021	0.020
**150**	b	89	f	93	5.4	16	0.751526	0.617147	Left	0.490	0.490	0.863	0.400	0.250	0.250	0.020	0.025	0.023
**151**	c	89	f	158	5.3	28.1	0.654047	0.502474	Right	0.340	0.340	0.616	0.400	0.250	0.250	0.013	0.017	0.015
**152**	c	89	f	88	4.1	18.4	0.654047	0.473221	Right	0.305	0.305	0.558	0.400	0.250	0.250	0.012	0.015	0.014
**153**	c	89	f	110	5.2	20.1	0.647507	0.445587	Right	0.274	0.274	0.506	0.400	0.250	0.250	0.011	0.013	0.012
**154**	c	89	f	115	5.3	20.5	0.660588	0.440525	Right	0.269	0.269	0.496	0.400	0.250	0.250	0.010	0.013	0.012
**155**	c	89	f	158	5.3	28.1	0.701697	0.567317	Left	0.422	0.422	0.752	0.400	0.250	0.250	0.017	0.021	0.020
**156**	c	89	f	88	4.1	18.4	0.701697	0.567317	Left	0.422	0.422	0.752	0.400	0.250	0.250	0.017	0.021	0.020
**157**	c	89	f	110	5.2	20.1	0.599857	0.465477	Left	0.297	0.297	0.543	0.400	0.250	0.250	0.012	0.014	0.013
**158**	c	89	f	115	5.3	20.5	0.708238	0.573858	Left	0.431	0.431	0.766	0.400	0.250	0.250	0.017	0.022	0.020
**159**	c	90	f	156	5.4	26.8	0.623631	0.416044	Right	0.243	0.243	0.452	0.400	0.250	0.250	0.009	0.011	0.011
**160**	c	90	f	130	5.6	21	0.629995	0.402268	Right	0.229	0.229	0.428	0.400	0.250	0.250	0.009	0.011	0.010
**161**	c	90	f	156	5.4	26.8	0.671281	0.536901	Left	0.382	0.382	0.687	0.400	0.250	0.250	0.015	0.019	0.018
**162**	c	90	f	130	5.6	21	0.677645	0.543265	Left	0.391	0.391	0.700	0.400	0.250	0.250	0.016	0.020	0.018
**163**	c	91	f	152	5.7	23.8	0.636358	0.404759	Right	0.231	0.231	0.432	0.400	0.250	0.250	0.009	0.011	0.010

**164**	c	91	f	152	5.7	23.8	0.588708	0.454329	Left	0.284	0.284	0.522	0.400	0.250	0.250	0.011	0.014	0.013
**165**	c	95	f	94	5.2	17.3	0.600981	0.351331	Right	0.180	0.180	0.342	0.400	0.250	0.250	0.007	0.008	0.008
**166**	c	95	f	115	5.3	20.4	0.588961	0.355063	Right	0.183	0.183	0.348	0.400	0.250	0.250	0.007	0.008	0.008
**167**	c	95	f	94	5.2	17.3	0.553331	0.418951	Left	0.246	0.246	0.457	0.400	0.250	0.250	0.009	0.012	0.011
**168**	c	95	f	115	5.3	20.4	0.541311	0.406931	Left	0.233	0.233	0.436	0.400	0.250	0.250	0.009	0.011	0.010
**3**	c	65	m	164	5.10.	23.9	1.179845	0.958491	Right	1.073	1.073	1.776	0.400	0.250	0.250	0.049	0.061	0.056
**4**	c	65	m	164	5.10.	23.9	1.227495	1.093116	Left	1.356	1.356	2.203	0.400	0.250	0.250	0.063	0.079	0.073
**169**	c	26	m	150	5.11	21	1.33942	1.099911	Right	1.371	1.371	2.226	0.400	0.250	0.250	0.064	0.080	0.074
**170**	c	26	m	150	5.11	21	1.38707	1.25269	Left	1.728	1.728	2.755	0.400	0.250	0.250	0.083	0.104	0.096
**171**	b	34	m	173	5.7	27.1	1.494392	1.29863	Right	1.842	1.842	2.923	0.400	0.250	0.250	0.090	0.111	0.103
**172**	b	34	m	173	5.7	27.1	1.446742	1.312362	Left	1.877	1.877	2.974	0.400	0.250	0.250	0.091	0.114	0.105
**173**	b	45	m	345	6.2	44.3	1.410038	1.238871	Right	1.694	1.694	2.705	0.400	0.250	0.250	0.081	0.101	0.094
**174**	b	45	m	345	6.2	44.3	1.362388	1.228008	Left	1.668	1.668	2.667	0.400	0.250	0.250	0.080	0.100	0.092
**175**	c	45	m	191	5.9	28.3	1.241185	1.04591	Right	1.253	1.253	2.049	0.400	0.250	0.250	0.058	0.072	0.067
**176**	c	45	m	191	5.9	28.3	1.288835	1.154455	Left	1.494	1.494	2.410	0.400	0.250	0.250	0.071	0.088	0.082
**177**	c	46	m	192	5.7	30.1	1.237487	0.996625	Right	1.150	1.150	1.893	0.400	0.250	0.250	0.053	0.066	0.061
**178**	c	46	m	192	5.7	30.1	1.189837	1.055457	Left	1.274	1.274	2.080	0.400	0.250	0.250	0.059	0.074	0.068
**179**	b	48	m	443	6.6	51	1.402525	1.354875	Right	1.987	1.987	3.133	0.400	0.250	0.250	0.097	0.121	0.112
**180**	b	48	m	443	6.6	51	1.184625	1.220495	Left	1.650	1.650	2.640	0.400	0.250	0.250	0.079	0.098	0.091
**181**	c	50	m	338	6.2	43.4	1.242518	1.020299	Right	1.199	1.199	1.968	0.400	0.250	0.250	0.055	0.069	0.064
**182**	c	50	m	338	6.2	43.4	1.194868	1.060488	Left	1.284	1.284	2.097	0.400	0.250	0.250	0.060	0.074	0.069
**183**	c	51	m	295	6.4	35.9	1.207976	0.988323	Right	1.133	1.133	1.868	0.400	0.250	0.250	0.052	0.065	0.060
**184**	c	51	m	200	6.2	25.7	1.207976	1.015015	Right	1.188	1.188	1.951	0.400	0.250	0.250	0.055	0.068	0.063
**185**	c	51	m	167	5.4	28.8	1.242518	1.054656	Right	1.272	1.272	2.078	0.400	0.250	0.250	0.059	0.074	0.068
**186**	c	51	m	295	6.4	35.9	1.255626	1.121246	Left	1.418	1.418	2.297	0.400	0.250	0.250	0.067	0.083	0.077
**187**	c	51	m	200	6.2	25.7	1.255626	1.121246	Left	1.418	1.418	2.297	0.400	0.250	0.250	0.067	0.083	0.077
**188**	c	51	m	167	5.4	28.8	1.194868	1.060488	Left	1.284	1.284	2.097	0.400	0.250	0.250	0.060	0.074	0.069
**189**	c	52	m	178	6	24.5	1.199672	1.038087	Right	1.237	1.237	2.024	0.400	0.250	0.250	0.057	0.071	0.066

**190**	c	52	m	178	6	24.5	1.247322	1.112942	Left	1.400	1.400	2.269	0.400	0.250	0.250	0.066	0.082	0.076
**191**	b	53	m	195	4	59.5	1.374138	1.217924	Right	1.643	1.643	2.631	0.400	0.250	0.250	0.079	0.098	0.091
**192**	b	53	m	195	4	59.5	1.421788	1.287408	Left	1.814	1.814	2.881	0.400	0.250	0.250	0.088	0.110	0.101
**193**	c	53	m	181	5.11	25.2	1.224155	1.033853	Right	1.228	1.228	2.011	0.400	0.250	0.250	0.057	0.071	0.065
**194**	c	53	m	171	5.7	26.9	1.211914	1.059576	Right	1.283	1.283	2.094	0.400	0.250	0.250	0.060	0.074	0.069
**195**	c	53	m	181	5.11	25.2	1.176505	1.042126	Left	1.245	1.245	2.037	0.400	0.250	0.250	0.058	0.072	0.066
**196**	c	53	m	171	5.7	26.9	1.164264	1.029884	Left	1.219	1.219	1.998	0.400	0.250	0.250	0.056	0.070	0.065
**197**	c	54	m	208	5.10.	29.9	1.22864	0.998146	Right	1.153	1.153	1.898	0.400	0.250	0.250	0.053	0.066	0.061
**198**	c	54	m	220	6.2	28.3	1.234723	1.00428	Right	1.166	1.166	1.917	0.400	0.250	0.250	0.054	0.067	0.062
**199**	c	54	m	181	5.9	26.8	1.235574	1.016166	Right	1.191	1.191	1.955	0.400	0.250	0.250	0.055	0.068	0.063
**200**	c	54	m	208	5.10.	29.9	1.18099	1.046611	Left	1.255	1.255	2.052	0.400	0.250	0.250	0.058	0.072	0.067
**201**	c	54	m	220	6.2	28.3	1.282373	1.147993	Left	1.479	1.479	2.388	0.400	0.250	0.250	0.070	0.087	0.081
**202**	c	54	m	181	5.9	26.8	1.187924	1.053545	Left	1.270	1.270	2.074	0.400	0.250	0.250	0.059	0.073	0.068
**203**	b	55	m	177	6	24.1	1.216476	0.973821	Right	1.104	1.104	1.823	0.400	0.250	0.250	0.050	0.063	0.058
**204**	b	55	m	189	5.6	30.6	1.363481	1.222112	Right	1.653	1.653	2.646	0.400	0.250	0.250	0.079	0.099	0.091
**205**	b	55	m	177	6	24.1	1.264126	1.129746	Left	1.438	1.438	2.326	0.400	0.250	0.250	0.068	0.084	0.078
**206**	b	55	m	189	5.6	30.6	1.411131	1.276751	Left	1.787	1.787	2.842	0.400	0.250	0.250	0.087	0.108	0.100
**207**	c	55	m	209	5.8	31.8	1.192146	1.026605	Right	1.212	1.212	1.988	0.400	0.250	0.250	0.056	0.070	0.064
**208**	c	55	m	209	5.8	31.8	1.239796	1.105416	Left	1.383	1.383	2.244	0.400	0.250	0.250	0.065	0.081	0.075
**209**	b	57	m	170	5.8	25.9	1.319786	1.166825	Right	1.523	1.523	2.452	0.400	0.250	0.250	0.072	0.090	0.083
**210**	b	57	m	170	5.8	25.9	1.272136	1.137757	Left	1.456	1.456	2.353	0.400	0.250	0.250	0.069	0.086	0.079
**211**	c	57	m	158	5.8	24	1.346721	1.209611	Right	1.623	1.623	2.601	0.400	0.250	0.250	0.078	0.097	0.090
**212**	c	57	m	198	5.5	33	1.189006	0.945319	Right	1.047	1.047	1.736	0.400	0.250	0.250	0.047	0.059	0.055
**213**	c	57	m	243	6	33	1.221972	1.013767	Right	1.186	1.186	1.947	0.400	0.250	0.250	0.055	0.068	0.063
**214**	c	57	m	158	5.8	24	1.299071	1.164691	Left	1.518	1.518	2.445	0.400	0.250	0.250	0.072	0.090	0.083
**215**	c	57	m	198	5.5	33	1.236656	1.102276	Left	1.376	1.376	2.234	0.400	0.250	0.250	0.065	0.080	0.074
**216**	c	57	m	243	6	33	1.269622	1.135243	Left	1.450	1.450	2.344	0.400	0.250	0.250	0.068	0.085	0.079
**217**	c	58	m	205	6	24.8	1.232629	1.015637	Right	1.189	1.189	1.953	0.400	0.250	0.250	0.055	0.068	0.063

**218**	c	58	m	190	5.6	30.7	1.221972	1.008722	Right	1.175	1.175	1.931	0.400	0.250	0.250	0.054	0.067	0.062
**219**	c	58	m	205	6	24.8	1.280279	1.145899	Left	1.474	1.474	2.380	0.400	0.250	0.250	0.070	0.087	0.080
**220**	c	58	m	190	5.6	30.7	1.174322	1.039943	Left	1.241	1.241	2.030	0.400	0.250	0.250	0.057	0.072	0.066
**221**	c	59	m	178	5.10.	25.6	1.203914	1.008737	Right	1.175	1.175	1.931	0.400	0.250	0.250	0.054	0.067	0.062
**222**	c	59	m	200	5.10.	28.7	1.179835	0.923566	Right	1.004	1.004	1.671	0.400	0.250	0.250	0.045	0.056	0.052
**223**	c	59	m	143	5.9	21.2	1.215953	1.088681	Right	1.346	1.346	2.189	0.400	0.250	0.250	0.063	0.078	0.073
**224**	c	59	m	178	5.10.	25.6	1.251564	1.117184	Left	1.409	1.409	2.283	0.400	0.250	0.250	0.066	0.083	0.076
**225**	c	59	m	200	5.10.	28.7	1.227485	1.093106	Left	1.356	1.356	2.203	0.400	0.250	0.250	0.063	0.079	0.073
**226**	c	59	m	143	5.9	21.2	1.263603	1.129223	Left	1.436	1.436	2.324	0.400	0.250	0.250	0.068	0.084	0.078
**227**	b	60	m	270	6	36.6	1.329776	1.141377	Right	1.464	1.464	2.365	0.400	0.250	0.250	0.069	0.086	0.080
**228**	b	60	m	270	6	36.6	1.282126	1.147746	Left	1.479	1.479	2.387	0.400	0.250	0.250	0.070	0.087	0.081
**229**	c	60	m	204	5.5	34	1.186058	0.969525	Right	1.095	1.095	1.810	0.400	0.250	0.250	0.050	0.062	0.058
**230**	c	60	m	150	5.8	22.9	1.191875	0.958677	Right	1.073	1.073	1.777	0.400	0.250	0.250	0.049	0.061	0.056
**231**	c	60	m	166	6	22.3	1.216009	1.000046	Right	1.157	1.157	1.904	0.400	0.250	0.250	0.053	0.066	0.061
**232**	c	60	m	204	5.5	34	1.138408	1.004028	Left	1.165	1.165	1.917	0.400	0.250	0.250	0.054	0.067	0.062
**233**	c	60	m	150	5.8	22.9	1.239525	1.105145	Left	1.382	1.382	2.243	0.400	0.250	0.250	0.065	0.081	0.075
**234**	c	60	m	166	6	22.3	1.263659	1.129279	Left	1.437	1.437	2.324	0.400	0.250	0.250	0.068	0.084	0.078
**235**	c	61	m	169	5.8	25.7	1.198038	0.986214	Right	1.129	1.129	1.861	0.400	0.250	0.250	0.052	0.064	0.060
**236**	c	61	m	190	5.6	30.7	1.174077	0.980775	Right	1.118	1.118	1.844	0.400	0.250	0.250	0.051	0.064	0.059
**237**	c	61	m	253	5.11	35.3	1.165986	0.923287	Right	1.004	1.004	1.670	0.400	0.250	0.250	0.045	0.056	0.052
**238**	c	61	m	255	6	34.6	1.210018	0.967636	Right	1.091	1.091	1.804	0.400	0.250	0.250	0.050	0.062	0.057
**239**	c	61	m	187	5.10.	26.8	1.188038	0.943643	Right	1.043	1.043	1.731	0.400	0.250	0.250	0.047	0.059	0.054
**240**	c	61	m	150	5.8	22.8	1.188782	0.952082	Right	1.060	1.060	1.757	0.400	0.250	0.250	0.048	0.060	0.055
**241**	c	61	m	169	5.8	25.7	1.150388	1.016008	Left	1.190	1.190	1.954	0.400	0.250	0.250	0.055	0.068	0.063
**242**	c	61	m	190	5.6	30.7	1.221727	1.087347	Left	1.343	1.343	2.184	0.400	0.250	0.250	0.063	0.078	0.072
**243**	c	61	m	253	5.11	35.3	1.118336	0.983957	Left	1.124	1.124	1.854	0.400	0.250	0.250	0.051	0.064	0.059
**244**	c	61	m	255	6	34.6	1.257668	1.123289	Left	1.423	1.423	2.304	0.400	0.250	0.250	0.067	0.083	0.077
**245**	c	61	m	187	5.10.	26.8	1.140388	1.006008	Left	1.169	1.169	1.923	0.400	0.250	0.250	0.054	0.067	0.062

**246**	c	61	m	150	5.8	22.8	1.141132	1.006752	Left	1.171	1.171	1.925	0.400	0.250	0.250	0.054	0.067	0.062
**247**	c	62	m	239	5.10.	34.3	1.213271	0.977302	Right	1.111	1.111	1.834	0.400	0.250	0.250	0.051	0.063	0.058
**248**	c	62	m	329	6.4	40.1	1.165986	0.953512	Right	1.063	1.063	1.761	0.400	0.250	0.250	0.048	0.060	0.056
**249**	c	62	m	194	5.10.	27.8	1.177884	0.952534	Right	1.061	1.061	1.758	0.400	0.250	0.250	0.048	0.060	0.056
**250**	c	62	m	224	6	30.5	1.199903	0.971957	Right	1.100	1.100	1.817	0.400	0.250	0.250	0.050	0.062	0.058
**251**	c	62	m	319	6.4	38.8	1.198723	0.958742	Right	1.073	1.073	1.777	0.400	0.250	0.250	0.049	0.061	0.056
**252**	c	62	m	239	5.10.	34.3	1.165621	1.031242	Left	1.222	1.222	2.003	0.400	0.250	0.250	0.056	0.070	0.065
**253**	c	62	m	329	6.4	40.1	1.213636	1.079257	Left	1.325	1.325	2.158	0.400	0.250	0.250	0.062	0.077	0.071
**254**	c	62	m	194	5.10.	27.8	1.225534	1.091154	Left	1.351	1.351	2.197	0.400	0.250	0.250	0.063	0.079	0.073
**255**	c	62	m	224	6	30.5	1.247553	1.113173	Left	1.400	1.400	2.270	0.400	0.250	0.250	0.066	0.082	0.076
**256**	c	62	m	319	6.4	38.8	1.151073	1.016693	Left	1.192	1.192	1.956	0.400	0.250	0.250	0.055	0.068	0.063
**257**	b	63	m	243	6.2	31.3	1.2716	1.069921	Right	1.305	1.305	2.127	0.400	0.250	0.250	0.061	0.076	0.070
**258**	b	63	m	243	6.2	31.3	1.31925	1.18487	Left	1.565	1.565	2.515	0.400	0.250	0.250	0.075	0.093	0.086
**259**	c	63	m	354	6.4	43.1	1.189782	0.950174	Right	1.056	1.056	1.751	0.400	0.250	0.250	0.048	0.060	0.055
**260**	c	63	m	210	5.8	31.9	1.168047	0.962836	Right	1.082	1.082	1.789	0.400	0.250	0.250	0.049	0.061	0.057
**261**	c	63	m	174	5.5	29	1.20168	1.041845	Right	1.245	1.245	2.036	0.400	0.250	0.250	0.058	0.072	0.066
**262**	c	63	m	244	6.1	32.2	1.189582	0.955743	Right	1.067	1.067	1.768	0.400	0.250	0.250	0.049	0.060	0.056
**263**	c	63	m	354	6.4	43.1	1.237432	1.103052	Left	1.378	1.378	2.236	0.400	0.250	0.250	0.065	0.080	0.074
**264**	c	63	m	210	5.8	31.9	1.120397	0.986017	Left	1.128	1.128	1.861	0.400	0.250	0.250	0.052	0.064	0.059
**265**	c	63	m	174	5.5	29	1.24933	1.11495	Left	1.404	1.404	2.276	0.400	0.250	0.250	0.066	0.082	0.076
**266**	c	63	m	244	6.1	32.2	1.237232	1.102852	Left	1.377	1.377	2.236	0.400	0.250	0.250	0.065	0.080	0.074
**267**	b	64	m	139	5.7	21.9	1.297551	1.111549	Right	1.397	1.397	2.265	0.400	0.250	0.250	0.066	0.082	0.076
**268**	b	64	m	139	5.7	21.9	1.249901	1.115521	Left	1.406	1.406	2.278	0.400	0.250	0.250	0.066	0.082	0.076
**269**	c	64	m	179	5.9	26.4	1.197543	0.989956	Right	1.136	1.136	1.873	0.400	0.250	0.250	0.052	0.065	0.060
**270**	c	64	m	200	5.5	33.4	1.179745	1.061953	Right	1.288	1.288	2.101	0.400	0.250	0.250	0.060	0.075	0.069
**271**	c	64	m	145	5.6	23.4	1.178845	0.990054	Right	1.137	1.137	1.873	0.400	0.250	0.250	0.052	0.065	0.060
**272**	c	64	m	179	5.9	26.4	1.149893	1.015513	Left	1.189	1.189	1.953	0.400	0.250	0.250	0.055	0.068	0.063
**273**	c	64	m	200	5.5	33.4	1.227395	1.093016	Left	1.355	1.355	2.203	0.400	0.250	0.250	0.063	0.079	0.073

**274**	c	64	m	145	5.6	23.4	1.131195	0.996816	Left	1.150	1.150	1.894	0.400	0.250	0.250	0.053	0.066	0.061
**275**	b	65	m	144	5.9	21.3	1.255719	1.056246	Right	1.275	1.275	2.083	0.400	0.250	0.250	0.059	0.074	0.068
**276**	b	65	m	207	5.9	30.7	1.268532	1.000648	Right	1.158	1.158	1.906	0.400	0.250	0.250	0.053	0.066	0.061
**277**	b	65	m	144	5.9	21.3	1.303369	1.168989	Left	1.528	1.528	2.460	0.400	0.250	0.250	0.073	0.090	0.084
**278**	b	65	m	207	5.9	30.7	1.220882	1.086502	Left	1.341	1.341	2.182	0.400	0.250	0.250	0.063	0.078	0.072
**279**	c	65	m	161	5.10.	23.1	1.156248	0.958017	Right	1.072	1.072	1.775	0.400	0.250	0.250	0.049	0.061	0.056
**280**	c	65	m	205	6.1	27.1	1.189578	0.974936	Right	1.106	1.106	1.826	0.400	0.250	0.250	0.050	0.063	0.058
**281**	c	65	m	143	5.6	23.1	1.178845	1.037456	Right	1.235	1.235	2.022	0.400	0.250	0.250	0.057	0.071	0.066
**282**	c	65	m	247	5.9	36.5	1.177745	1.036356	Right	1.233	1.233	2.019	0.400	0.250	0.250	0.057	0.071	0.066
**283**	c	65	m	161	5.10.	23.1	1.108598	0.974219	Left	1.104	1.104	1.824	0.400	0.250	0.250	0.050	0.063	0.058
**284**	c	65	m	205	6.1	27.1	1.237228	1.102848	Left	1.377	1.377	2.236	0.400	0.250	0.250	0.065	0.080	0.074
**285**	c	65	m	143	5.6	23.1	1.131195	0.996816	Left	1.150	1.150	1.894	0.400	0.250	0.250	0.053	0.066	0.061
**286**	c	65	m	247	5.9	36.5	1.130095	0.995716	Left	1.148	1.148	1.891	0.400	0.250	0.250	0.053	0.066	0.061
**287**	b	66	m	199	5.8	30.3	1.279847	1.058581	Right	1.280	1.280	2.090	0.400	0.250	0.250	0.059	0.074	0.069
**288**	b	66	m	199	5.8	30.3	1.232197	1.097817	Left	1.366	1.366	2.219	0.400	0.250	0.250	0.064	0.080	0.074
**289**	c	66	m	181	5.6	30.3	1.169693	0.916258	Right	0.990	0.990	1.650	0.400	0.250	0.250	0.045	0.056	0.051
**290**	c	66	m	214	6.2	27.5	1.157996	0.974575	Right	1.105	1.105	1.825	0.400	0.250	0.250	0.050	0.063	0.058
**291**	c	66	m	181	5.6	30.3	1.122043	0.987663	Left	1.132	1.132	1.866	0.400	0.250	0.250	0.052	0.064	0.060
**292**	c	66	m	214	6.2	27.5	1.205646	1.071266	Left	1.308	1.308	2.132	0.400	0.250	0.250	0.061	0.076	0.070
**293**	b	67	m	123	5.9	18.2	1.281346	1.155527	Right	1.497	1.497	2.413	0.400	0.250	0.250	0.071	0.088	0.082
**294**	b	67	m	123	5.9	18.2	1.233696	1.099316	Left	1.369	1.369	2.224	0.400	0.250	0.250	0.064	0.080	0.074
**295**	c	67	m	283	6	38.9	1.146299	0.923225	Right	1.004	1.004	1.670	0.400	0.250	0.250	0.045	0.056	0.052
**296**	c	67	m	224	6.3	28	1.191644	0.943766	Right	1.044	1.044	1.732	0.400	0.250	0.250	0.047	0.059	0.054
**297**	c	67	m	118	6.1	15.6	1.20168	1.066754	Right	1.298	1.298	2.117	0.400	0.250	0.250	0.060	0.075	0.070
**298**	c	67	m	234	5.11	33.1	1.173014	1.043512	Right	1.248	1.248	2.042	0.400	0.250	0.250	0.058	0.072	0.067
**299**	c	67	m	158	5.8	24.1	1.179445	0.943496	Right	1.043	1.043	1.731	0.400	0.250	0.250	0.047	0.059	0.054
**300**	c	67	m	182	5.10.	26.1	1.169193	0.995399	Right	1.148	1.148	1.890	0.400	0.250	0.250	0.053	0.066	0.061

**301**	c	67	m	283	6	38.9	1.098649	0.96427	Left	1.084	1.084	1.794	0.400	0.250	0.250	0.049	0.061	0.057
**302**	c	67	m	224	6.3	28	1.239294	1.104914	Left	1.382	1.382	2.242	0.400	0.250	0.250	0.065	0.081	0.075
**303**	c	67	m	118	6.1	15.6	1.15403	1.01965	Left	1.198	1.198	1.966	0.400	0.250	0.250	0.055	0.069	0.064
**304**	c	67	m	234	5.11	33.1	1.220664	1.086284	Left	1.341	1.341	2.181	0.400	0.250	0.250	0.063	0.078	0.072
**305**	c	67	m	158	5.8	24.1	1.131795	0.997416	Left	1.152	1.152	1.896	0.400	0.250	0.250	0.053	0.066	0.061
**306**	c	67	m	182	5.10.	26.1	1.121543	0.987163	Left	1.131	1.131	1.864	0.400	0.250	0.250	0.052	0.064	0.060
**307**	m	67	m	197	5.9	29.1	1.218434	1.012343	Right	1.183	1.183	1.943	0.400	0.250	0.250	0.054	0.068	0.063
**308**	m	67	m	197	5.9	29.1	1.170784	1.036404	Left	1.233	1.233	2.019	0.400	0.250	0.250	0.057	0.071	0.066
**309**	b	68	m	270	5.7	42.3	1.267175	1.063216	Right	1.290	1.290	2.105	0.400	0.250	0.250	0.060	0.075	0.069
**310**	b	68	m	270	5.7	42.3	1.314825	1.180445	Left	1.554	1.554	2.499	0.400	0.250	0.250	0.074	0.092	0.085
**311**	c	68	m	180	6.1	23.9	1.152273	0.914316	Right	0.986	0.986	1.644	0.400	0.250	0.250	0.044	0.055	0.051
**312**	c	68	m	208	5.7	32.6	1.129227	0.866513	Right	0.897	0.897	1.505	0.400	0.250	0.250	0.040	0.050	0.046
**313**	c	68	m	335	5.11	46.8	1.115372	0.889526	Right	0.939	0.939	1.571	0.400	0.250	0.250	0.042	0.052	0.048
**314**	c	68	m	133	5.3	23.6	1.18139	0.962249	Right	1.080	1.080	1.788	0.400	0.250	0.250	0.049	0.061	0.057
**315**	c	68	m	166	5.7	26	1.169557	1.003025	Right	1.163	1.163	1.913	0.400	0.250	0.250	0.053	0.067	0.062
**316**	c	68	m	196	5.7	30.8	1.169493	0.948159	Right	1.052	1.052	1.745	0.400	0.250	0.250	0.048	0.059	0.055
**317**	c	68	m	244	5.11	34.1	1.152473	1.018126	Right	1.195	1.195	1.961	0.400	0.250	0.250	0.055	0.069	0.063
**318**	c	68	m	180	6.1	23.9	1.104623	0.970243	Left	1.096	1.096	1.812	0.400	0.250	0.250	0.050	0.062	0.058
**319**	c	68	m	208	5.7	32.6	1.081577	0.947198	Left	1.051	1.051	1.742	0.400	0.250	0.250	0.048	0.059	0.055
**320**	c	68	m	335	5.11	46.8	1.163022	1.028642	Left	1.217	1.217	1.994	0.400	0.250	0.250	0.056	0.070	0.065
**321**	c	68	m	133	5.3	23.6	1.22904	1.09466	Left	1.359	1.359	2.208	0.400	0.250	0.250	0.064	0.079	0.073
**322**	c	68	m	166	5.7	26	1.121907	0.987527	Left	1.131	1.131	1.865	0.400	0.250	0.250	0.052	0.064	0.060
**323**	c	68	m	196	5.7	30.8	1.217143	1.082763	Left	1.333	1.333	2.169	0.400	0.250	0.250	0.062	0.078	0.072
**324**	c	68	m	244	5.11	34.1	1.200123	1.065743	Left	1.296	1.296	2.114	0.400	0.250	0.250	0.060	0.075	0.069
**325**	b	69	m	297	6.5	35.3	1.252634	1.139363	Right	1.459	1.459	2.358	0.400	0.250	0.250	0.069	0.086	0.079
**326**	b	69	m	297	6.5	35.3	1.300284	1.165905	Left	1.521	1.521	2.449	0.400	0.250	0.250	0.072	0.090	0.083
**327**	c	69	m	195	4	59.6	1.138134	0.961355	Right	1.079	1.079	1.785	0.400	0.250	0.250	0.049	0.061	0.057
**328**	c	69	m	175	5.11	24.5	1.129227	0.964344	Right	1.085	1.085	1.794	0.400	0.250	0.250	0.049	0.061	0.057
**329**	c	69	m	265	6.1	35	1.14075	0.937612	Right	1.032	1.032	1.713	0.400	0.250	0.250	0.047	0.058	0.054

**330**	c	69	m	177	5.10.	25.5	1.163796	0.93753	Right	1.031	1.031	1.713	0.400	0.250	0.250	0.047	0.058	0.054
**331**	c	69	m	195	5.9	28.8	1.187239	1.038669	Right	1.238	1.238	2.026	0.400	0.250	0.250	0.057	0.071	0.066
**332**	c	69	m	192	5.1	26.8	1.171862	0.937259	Right	1.031	1.031	1.712	0.400	0.250	0.250	0.047	0.058	0.054
**333**	c	69	m	195	4	59.6	1.090484	0.956104	Left	1.068	1.068	1.769	0.400	0.250	0.250	0.049	0.060	0.056
**334**	c	69	m	175	5.11	24.5	1.081577	0.947198	Left	1.051	1.051	1.742	0.400	0.250	0.250	0.048	0.059	0.055
**335**	c	69	m	265	6.1	35	1.1884	1.05402	Left	1.271	1.271	2.076	0.400	0.250	0.250	0.059	0.073	0.068
**336**	c	69	m	177	5.10.	25.5	1.211446	1.077066	Left	1.320	1.320	2.151	0.400	0.250	0.250	0.062	0.077	0.071
**337**	c	69	m	195	5.9	28.8	1.139589	1.005209	Left	1.168	1.168	1.920	0.400	0.250	0.250	0.054	0.067	0.062
**338**	c	69	m	192	5.1	26.8	1.124212	0.989832	Left	1.136	1.136	1.872	0.400	0.250	0.250	0.052	0.065	0.060
**339**	b	70	m	190	6.1	25.1	1.239388	1.001587	Right	1.160	1.160	1.909	0.400	0.250	0.250	0.053	0.066	0.061
**340**	b	70	m	190	6.1	25.1	1.191738	1.057359	Left	1.278	1.278	2.086	0.400	0.250	0.250	0.059	0.074	0.068
**341**	c	71	m	203	5.8	31.4	1.126753	0.973633	Right	1.103	1.103	1.822	0.400	0.250	0.250	0.050	0.063	0.058
**342**	c	71	m	218	5.9	32.2	1.155206	1.027043	Right	1.213	1.213	1.989	0.400	0.250	0.250	0.056	0.070	0.065
**343**	c	71	m	262	6.8	28.8	1.137134	0.888631	Right	0.938	0.938	1.569	0.400	0.250	0.250	0.042	0.052	0.048
**344**	c	71	m	208	5.11	29.1	1.151273	0.913516	Right	0.985	0.985	1.642	0.400	0.250	0.250	0.044	0.055	0.051
**345**	c	71	m	267	5.11	37.2	1.138634	1.009163	Right	1.176	1.176	1.933	0.400	0.250	0.250	0.054	0.067	0.062
**346**	c	71	m	203	5.8	31.4	1.079103	0.944723	Left	1.046	1.046	1.734	0.400	0.250	0.250	0.047	0.059	0.055
**347**	c	71	m	218	5.9	32.2	1.107556	0.973176	Left	1.102	1.102	1.821	0.400	0.250	0.250	0.050	0.063	0.058
**348**	c	71	m	262	6.8	28.8	1.184784	1.050404	Left	1.263	1.263	2.064	0.400	0.250	0.250	0.059	0.073	0.068
**349**	c	71	m	208	5.11	29.1	1.198923	1.064543	Left	1.293	1.293	2.110	0.400	0.250	0.250	0.060	0.075	0.069
**350**	c	71	m	267	5.11	37.2	1.090984	0.956604	Left	1.069	1.069	1.770	0.400	0.250	0.250	0.049	0.061	0.056
**351**	c	72	m	239	5.10.	34.4	1.10947	0.869536	Right	0.902	0.902	1.514	0.400	0.250	0.250	0.040	0.050	0.046
**352**	c	72	m	238	6	32.4	1.149516	0.965033	Right	1.086	1.086	1.796	0.400	0.250	0.250	0.049	0.062	0.057
**353**	c	72	m	178	5.7	28	1.139728	0.930023	Right	1.017	1.017	1.690	0.400	0.250	0.250	0.046	0.057	0.053
**354**	c	72	m	239	5.10.	34.4	1.06182	0.92744	Left	1.012	1.012	1.683	0.400	0.250	0.250	0.046	0.057	0.053
**355**	c	72	m	238	6	32.4	1.197166	1.062786	Left	1.289	1.289	2.104	0.400	0.250	0.250	0.060	0.075	0.069
**356**	c	72	m	178	5.7	28	1.092078	0.957698	Left	1.071	1.071	1.774	0.400	0.250	0.250	0.049	0.061	0.056
**357**	b	73	m	216	6.3	27	1.224293	0.988716	Right	1.134	1.134	1.869	0.400	0.250	0.250	0.052	0.065	0.060
**358**	b	73	m	216	6.3	27	1.176643	1.042263	Left	1.245	1.245	2.038	0.400	0.250	0.250	0.058	0.072	0.066

**359**	c	73	m	152	5.9	22.6	1.120677	0.880516	Right	0.922	0.922	1.545	0.400	0.250	0.250	0.041	0.051	0.047
**360**	c	73	m	190	5.7	29.9	1.098263	0.84088	Right	0.850	0.850	1.433	0.400	0.250	0.250	0.038	0.047	0.043
**361**	c	73	m	218	5.7	34.1	1.098263	0.853667	Right	0.873	0.873	1.469	0.400	0.250	0.250	0.039	0.048	0.045
**362**	c	73	m	228	6	30.9	1.098263	0.862296	Right	0.889	0.889	1.493	0.400	0.250	0.250	0.039	0.049	0.045
**363**	c	73	m	229	5.11	31.9	1.131884	0.908538	Right	0.975	0.975	1.627	0.400	0.250	0.250	0.044	0.055	0.051
**364**	c	73	m	180	5.9	26.6	1.123375	0.887798	Right	0.936	0.936	1.566	0.400	0.250	0.250	0.042	0.052	0.048
**365**	c	73	m	201	5.10.	29.3	1.137487	0.932969	Right	1.023	1.023	1.699	0.400	0.250	0.250	0.046	0.058	0.053
**366**	c	73	m	152	5.9	22.6	1.168327	1.033947	Left	1.228	1.228	2.011	0.400	0.250	0.250	0.057	0.071	0.065
**367**	c	73	m	190	5.7	29.9	1.050613	0.916233	Left	0.990	0.990	1.650	0.400	0.250	0.250	0.045	0.056	0.051
**368**	c	73	m	218	5.7	34.1	1.050613	0.916233	Left	0.990	0.990	1.650	0.400	0.250	0.250	0.045	0.056	0.051
**369**	c	73	m	228	6	30.9	1.050613	0.916233	Left	0.990	0.990	1.650	0.400	0.250	0.250	0.045	0.056	0.051
**370**	c	73	m	229	5.11	31.9	1.179534	1.045154	Left	1.252	1.252	2.047	0.400	0.250	0.250	0.058	0.072	0.067
**371**	c	73	m	180	5.9	26.6	1.075725	0.941345	Left	1.039	1.039	1.724	0.400	0.250	0.250	0.047	0.059	0.054
**372**	c	73	m	201	5.10.	29.3	1.089837	0.955457	Left	1.067	1.067	1.767	0.400	0.250	0.250	0.048	0.060	0.056
**373**	c	74	m	194	5.8	29.5	1.084638	0.946096	Right	1.048	1.048	1.739	0.400	0.250	0.250	0.048	0.059	0.055
**374**	c	74	m	196	5.6	31.6	1.095705	0.847469	Right	0.862	0.862	1.451	0.400	0.250	0.250	0.038	0.047	0.044
**375**	c	74	m	267	5.11	37.4	1.106673	0.876419	Right	0.915	0.915	1.534	0.400	0.250	0.250	0.041	0.051	0.047
**376**	c	74	m	194	5.8	29.5	1.132288	0.997908	Left	1.153	1.153	1.897	0.400	0.250	0.250	0.053	0.066	0.061
**377**	c	74	m	196	5.6	31.6	1.048055	0.913676	Left	0.985	0.985	1.642	0.400	0.250	0.250	0.044	0.055	0.051
**378**	c	74	m	267	5.11	37.4	1.059023	0.924643	Left	1.006	1.006	1.674	0.400	0.250	0.250	0.045	0.057	0.052
**379**	c	75	m	190	5.10.	27.3	1.117841	0.987062	Right	1.130	1.130	1.864	0.400	0.250	0.250	0.052	0.064	0.060
**380**	c	75	m	191	5.8	29.1	1.100313	0.975586	Right	1.107	1.107	1.828	0.400	0.250	0.250	0.051	0.063	0.058
**381**	c	75	m	190	5.10.	27.3	1.165491	1.031111	Left	1.222	1.222	2.002	0.400	0.250	0.250	0.056	0.070	0.065
**382**	c	75	m	191	5.8	29.1	1.052663	0.918283	Left	0.994	0.994	1.656	0.400	0.250	0.250	0.045	0.056	0.052
**383**	c	76	m	140	5.2	25.6	1.094892	0.897404	Right	0.954	0.954	1.594	0.400	0.250	0.250	0.043	0.053	0.049
**384**	c	76	m	109	5.8	16.6	1.101071	0.903426	Right	0.966	0.966	1.612	0.400	0.250	0.250	0.043	0.054	0.050
**385**	c	76	m	140	5.2	25.6	1.047242	0.912863	Left	0.984	0.984	1.640	0.400	0.250	0.250	0.044	0.055	0.051
**386**	c	76	m	109	5.8	16.6	1.053421	0.919042	Left	0.996	0.996	1.658	0.400	0.250	0.250	0.045	0.056	0.052

**387**	b	77	m	190	6.2	24.4	1.190958	0.993952	Right	1.145	1.145	1.885	0.400	0.250	0.250	0.052	0.065	0.060
**388**	b	77	m	190	6.2	24.4	1.143308	1.008928	Left	1.175	1.175	1.932	0.400	0.250	0.250	0.054	0.067	0.062
**389**	c	77	m	183	5.10.	26.3	1.084052	0.878947	Right	0.920	0.920	1.541	0.400	0.250	0.250	0.041	0.051	0.047
**390**	c	77	m	199	5.11	27.8	1.05912	0.804058	Right	0.785	0.785	1.331	0.400	0.250	0.250	0.034	0.043	0.040
**391**	c	77	m	175	5.7	27.4	1.036421	0.791589	Right	0.763	0.763	1.298	0.400	0.250	0.250	0.033	0.041	0.038
**392**	c	77	m	191	5.6	30.8	1.062371	0.889127	Right	0.939	0.939	1.570	0.400	0.250	0.250	0.042	0.052	0.048
**393**	c	77	m	164	5.6	22.6	1.073211	0.870024	Right	0.903	0.903	1.515	0.400	0.250	0.250	0.040	0.050	0.046
**394**	c	77	m	183	5.10.	26.3	1.131702	0.997322	Left	1.151	1.151	1.896	0.400	0.250	0.250	0.053	0.066	0.061
**395**	c	77	m	199	5.11	27.8	1.10677	0.97239	Left	1.101	1.101	1.819	0.400	0.250	0.250	0.050	0.063	0.058
**396**	c	77	m	175	5.7	27.4	1.084071	0.949691	Left	1.055	1.055	1.749	0.400	0.250	0.250	0.048	0.060	0.055
**397**	c	77	m	191	5.6	30.8	1.014721	0.880341	Left	0.922	0.922	1.545	0.400	0.250	0.250	0.041	0.051	0.047
**398**	c	77	m	164	5.6	22.6	1.025561	0.891181	Left	0.942	0.942	1.576	0.400	0.250	0.250	0.042	0.053	0.049
**399**	c	78	m	118	5.5	19.6	1.037937	0.825624	Right	0.823	0.823	1.391	0.400	0.250	0.250	0.036	0.045	0.042
**400**	c	78	m	118	5.5	19.6	1.085587	0.951207	Left	1.058	1.058	1.754	0.400	0.250	0.250	0.048	0.060	0.055
**401**	c	79	m	218	6.1	28.8	1.037937	0.837469	Right	0.844	0.844	1.423	0.400	0.250	0.250	0.037	0.046	0.043
**402**	c	79	m	218	6.1	28.8	1.085587	0.951207	Left	1.058	1.058	1.754	0.400	0.250	0.250	0.048	0.060	0.055
**403**	c	80	m	208	5.10.	29.9	1.026056	0.794807	Right	0.769	0.769	1.306	0.400	0.250	0.250	0.034	0.042	0.039
**404**	c	80	m	208	5.10.	29.9	1.073706	0.939327	Left	1.035	1.035	1.718	0.400	0.250	0.250	0.047	0.058	0.054
**405**	c	81	m	166	6	22.5	1.015692	0.789315	Right	0.759	0.759	1.292	0.400	0.250	0.250	0.033	0.041	0.038
**406**	c	81	m	225	6	30.5	1.002339	0.867971	Right	0.899	0.899	1.509	0.400	0.250	0.250	0.040	0.050	0.046
**407**	c	81	m	201	5.6	32.6	1.022589	0.797914	Right	0.774	0.774	1.315	0.400	0.250	0.250	0.034	0.042	0.039
**408**	c	81	m	166	6	22.5	0.968042	0.833662	Left	0.837	0.837	1.413	0.400	0.250	0.250	0.037	0.046	0.043
**409**	c	81	m	225	6	30.5	0.954689	0.82031	Left	0.813	0.813	1.376	0.400	0.250	0.250	0.036	0.044	0.041
**410**	c	81	m	201	5.6	32.6	1.070239	0.935859	Left	1.028	1.028	1.708	0.400	0.250	0.250	0.047	0.058	0.054
**411**	ai	82	m	153	5.6	25.1	0.911218	0.673302	Right	0.572	0.572	0.995	0.400	0.250	0.250	0.024	0.030	0.028
**412**	ai	82	m	153	5.6	25.1	0.863568	0.729188	Left	0.659	0.659	1.134	0.400	0.250	0.250	0.028	0.035	0.033
**413**	c	82	m	205	5.6	33.1	1.012464	0.769169	Right	0.725	0.725	1.238	0.400	0.250	0.250	0.031	0.039	0.036

**414**	c	82	m	160	5.10.	23.1	0.992215	0.837692	Right	0.844	0.844	1.424	0.400	0.250	0.250	0.037	0.046	0.043
**415**	c	82	m	205	5.6	33.1	0.964814	0.830434	Left	0.831	0.831	1.404	0.400	0.250	0.250	0.037	0.046	0.042
**416**	c	82	m	160	5.10.	23.1	1.039865	0.905485	Left	0.970	0.970	1.618	0.400	0.250	0.250	0.044	0.054	0.050
**417**	c	84	m	233	5.6	37.6	0.986234	0.868067	Right	0.899	0.899	1.510	0.400	0.250	0.250	0.040	0.050	0.046
**418**	c	84	m	195	5.7	30.6	0.966509	0.8357	Right	0.841	0.841	1.418	0.400	0.250	0.250	0.037	0.046	0.043
**419**	c	84	m	188	5.7	29.6	0.996096	0.757667	Right	0.706	0.706	1.208	0.400	0.250	0.250	0.030	0.038	0.035
**420**	c	84	m	233	5.6	37.6	0.938584	0.804204	Left	0.785	0.785	1.332	0.400	0.250	0.250	0.034	0.043	0.040
**421**	c	84	m	195	5.7	30.6	0.918859	0.784479	Left	0.751	0.751	1.279	0.400	0.250	0.250	0.033	0.041	0.038
**422**	c	84	m	188	5.7	29.6	0.948446	0.814066	Left	0.802	0.802	1.359	0.400	0.250	0.250	0.035	0.044	0.041
**423**	b	87	m	149	6.1	19.7	1.111095	0.952046	Right	1.060	1.060	1.757	0.400	0.250	0.250	0.048	0.060	0.055
**424**	b	87	m	149	6.1	19.7	1.158745	1.024365	Left	1.208	1.208	1.981	0.400	0.250	0.250	0.056	0.069	0.064
**425**	c	87	m	149	5.10.	21.4	1.009846	0.755526	Right	0.702	0.702	1.202	0.400	0.250	0.250	0.030	0.038	0.035
**426**	c	87	m	149	5.10.	21.4	0.962196	0.827816	Left	0.827	0.827	1.397	0.400	0.250	0.250	0.036	0.045	0.042
**427**	c	88	m	164	5.3	29.1	1.030455	0.831236	Right	0.833	0.833	1.406	0.400	0.250	0.250	0.037	0.046	0.042
**428**	c	88	m	164	5.3	29.1	1.078105	0.943725	Left	1.044	1.044	1.731	0.400	0.250	0.250	0.047	0.059	0.054
**429**	b	89	m	112	5.11	15.8	1.106327	0.978134	Right	1.112	1.112	1.836	0.400	0.250	0.250	0.051	0.063	0.059
**430**	b	89	m	112	5.11	15.8	1.153977	1.019597	Left	1.198	1.198	1.966	0.400	0.250	0.250	0.055	0.069	0.064
**431**	c	89	m	209	5.8	32.4	0.999036	0.761588	Right	0.713	0.713	1.218	0.400	0.250	0.250	0.031	0.038	0.035
**432**	c	89	m	209	5.8	32.4	1.046686	0.912306	Left	0.983	0.983	1.638	0.400	0.250	0.250	0.044	0.055	0.051
**433**	c	90	m	123	5.6	19.9	1.019424	0.82815	Right	0.827	0.827	1.398	0.400	0.250	0.250	0.036	0.045	0.042
**434**	c	90	m	123	5.6	19.9	1.067074	0.932694	Left	1.022	1.022	1.698	0.400	0.250	0.250	0.046	0.058	0.053
**435**	c	91	m	175	6	23.8	0.988225	0.752456	Right	0.697	0.697	1.194	0.400	0.250	0.250	0.030	0.037	0.035
**436**	c	91	m	175	6	23.8	1.035875	0.901496	Left	0.962	0.962	1.606	0.400	0.250	0.250	0.043	0.054	0.050
**437**	c	92	m	175	5.4	30	1.008393	0.84065	Right	0.849	0.849	1.432	0.400	0.250	0.250	0.038	0.047	0.043
**438**	c	92	m	175	5.4	30	0.960743	0.826364	Left	0.824	0.824	1.393	0.400	0.250	0.250	0.036	0.045	0.042

Supplementary materials B

The Statistical Analysis

Bivariate Fit of Density by Age

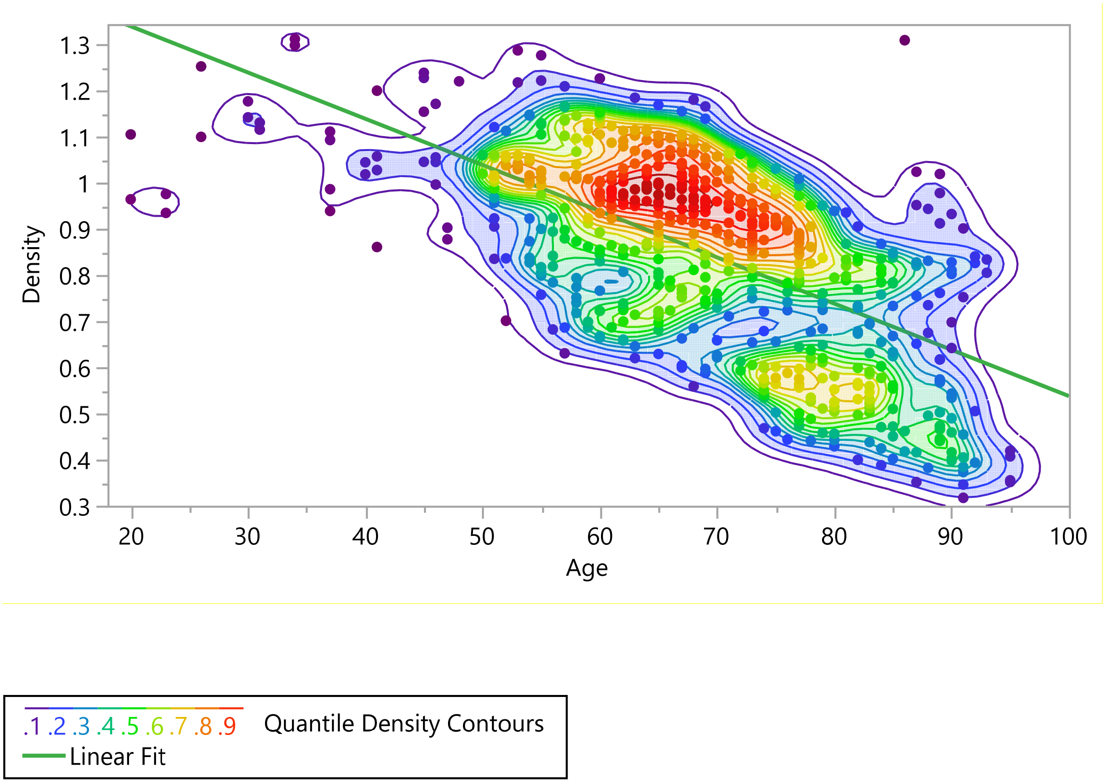

Summary Statistics

	Value	Lower 95%	Upper 95%	Signif. Prob
Correlation	-0.62399	-0.67004	-0.57317	<.0001*
Covariance	-1.76426			
Count	614			

Variable	Mean	Std Dev
Age	68.71987	13.266

Variable	Mean	Std Dev
Density	0.851499	0.213129

Quantile Density Contours

Variable	Kernel Std
Age	2.275177
Density	0.036553

Linear Fit

Density = 1.5404122 - 0.010025*Age

Summary of Fit

RSquare	0.389366
RSquare Adj	0.388369
Root Mean Square Error	0.166682
Mean of Response	0.851499
Observations (or Sum Wgts)	614

Analysis of Variance

Source	DF	Sum of Squares	Mean Square	F Ratio
Model	1	10.841885	10.8419	390.2376
Error	612	17.003059	0.0278	**Prob > F**
C. Total	613	27.844944		<.0001*

Parameter Estimates

Term	Estimate	Std Error	t Ratio	Prob>|t|
Intercept	1.5404122	0.035517	43.37	<.0001*
Age	-0.010025	0.000507	-19.75	<.0001*

Diagnostics Plots

Residual by Predicted Plot

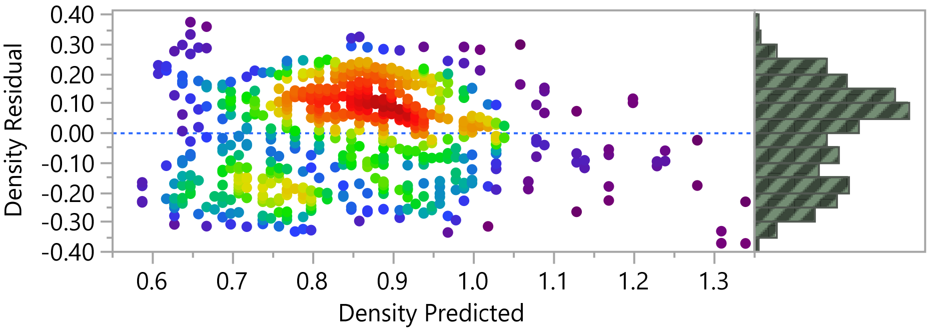

Actual by Predicted Plot

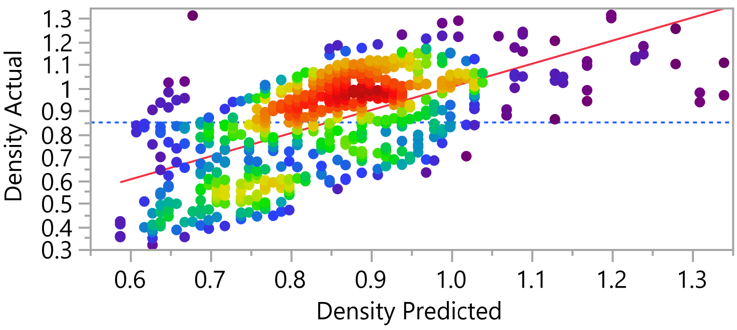

Residual by Row Plot

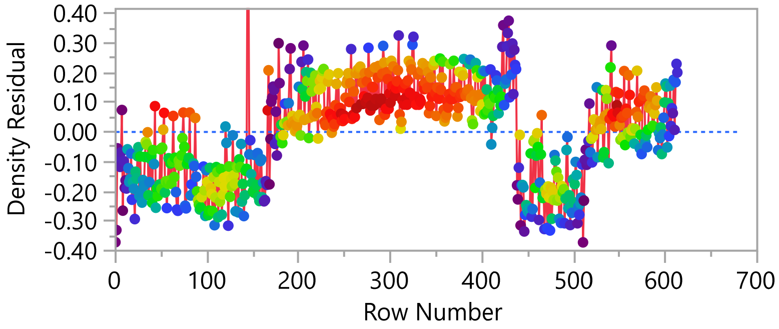

Residual by X Plot

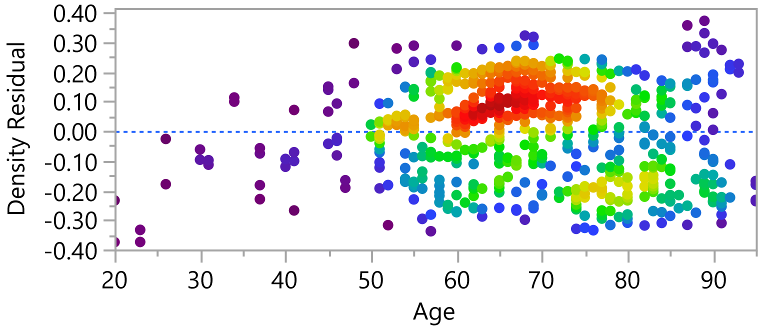

Residual Normal Quantile Plot

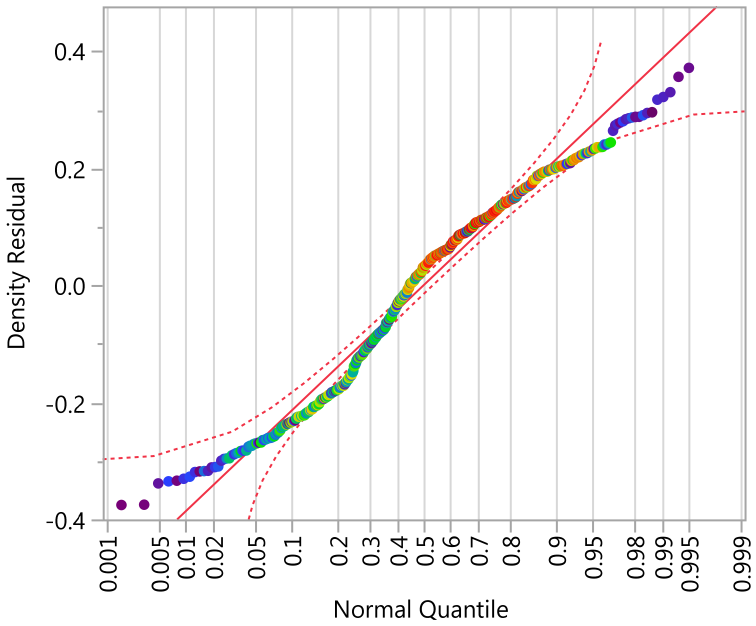

Oneway Analysis of Density By Gender

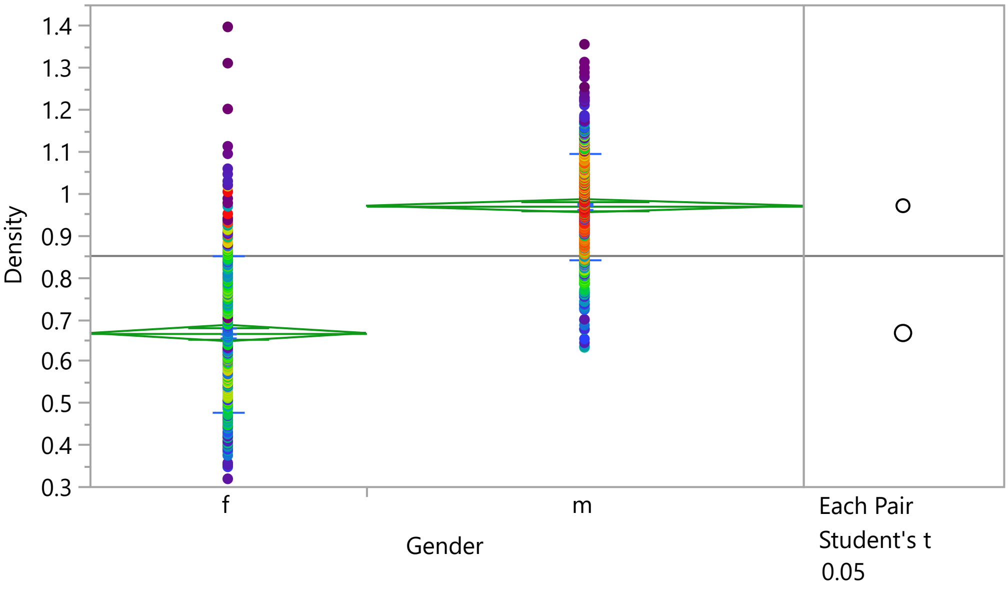

Oneway Anova

Summary of Fit

Rsquare	0.483592
Adj Rsquare	0.482748
Root Mean Square Error	0.153283
Mean of Response	0.851499
Observations (or Sum Wgts)	614

Pooled t Test

m-f

Assuming equal variances

Difference	0.303959	t Ratio	23.93969
Std Err Dif	0.012697	DF	612

Upper CL Dif	0.328893	Prob > |t|	<.0001*
Lower CL Dif	0.279024	Prob > t	<.0001*
Confidence	0.95	Prob < t	1.0000

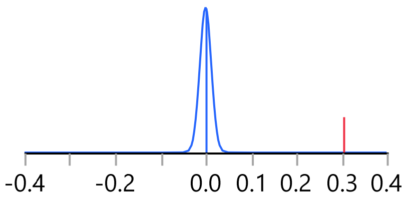

Analysis of Variance

Source	DF	Sum of Squares	Mean Square	F Ratio	Prob > F
Gender	1	13.465586	13.4656	573.1090	<.0001*
Error	612	14.379358	0.0235		
C. Total	613	27.844944			

Means for Oneway Anova

Level	Number	Mean	Std Error	Lower 95%	Upper 95%
f	238	0.665361	0.00994	0.64585	0.68487
m	376	0.969320	0.00790	0.95380	0.98484

Std Error uses a pooled estimate of error variance

Means and Std Deviations

Level	Number	Mean	Std Dev	Std Err Mean	Lower 95%	Upper 95%
f	238	0.6653613	0.187628	0.0121621	0.6414017	0.689321
m	376	0.9693199	0.1268695	0.0065428	0.9564548	0.9821851

t Test

m-f

Assuming unequal variances

Difference	0.303959	t Ratio	22.00951
Std Err Dif	0.013810	DF	374.22
Upper CL Dif	0.331114	Prob > |t|	<.0001*
Lower CL Dif	0.276803	Prob > t	<.0001*
Confidence	0.95	Prob < t	1.0000

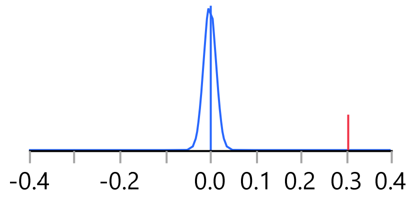

CDF Plot

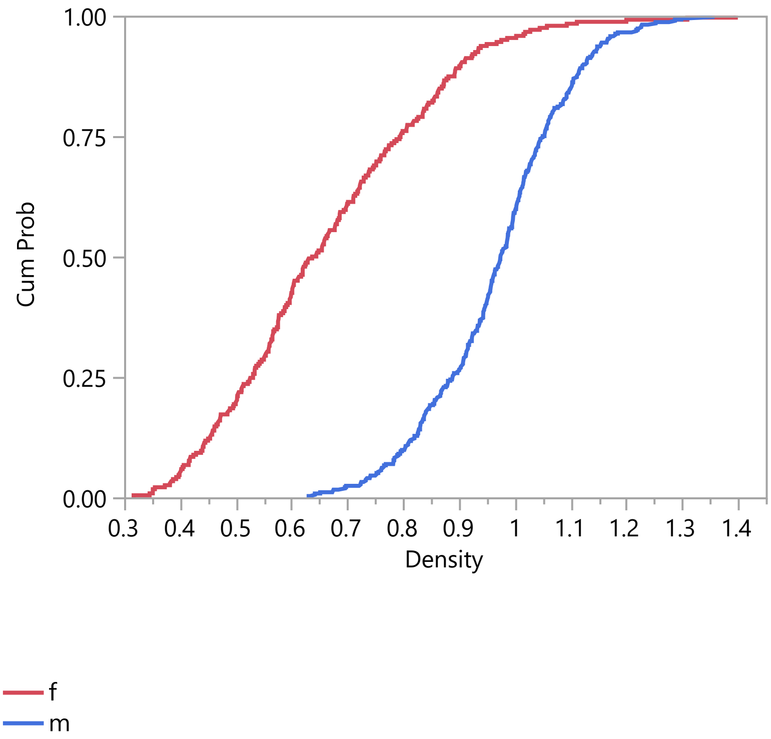

Compare Densities

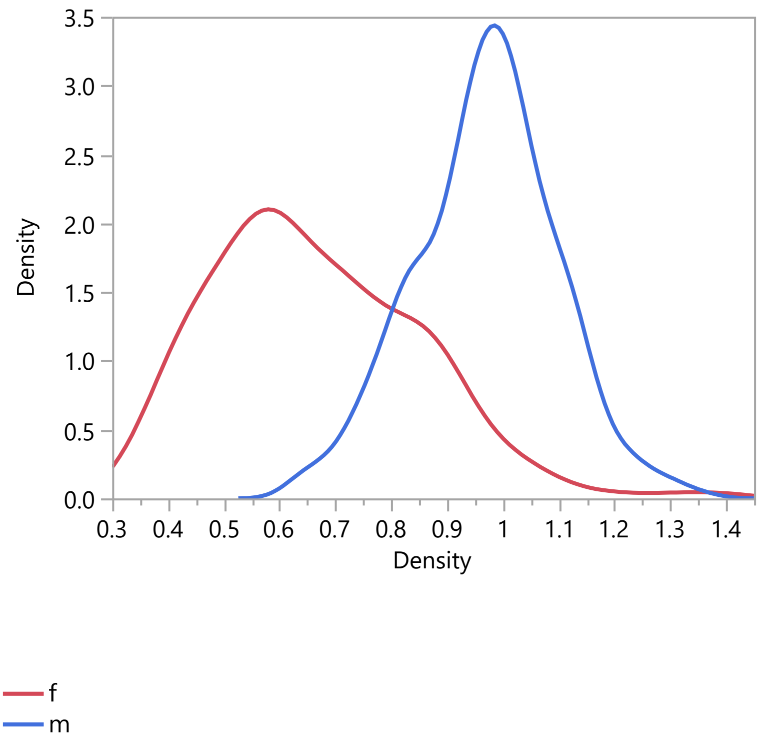

Composition of Densities

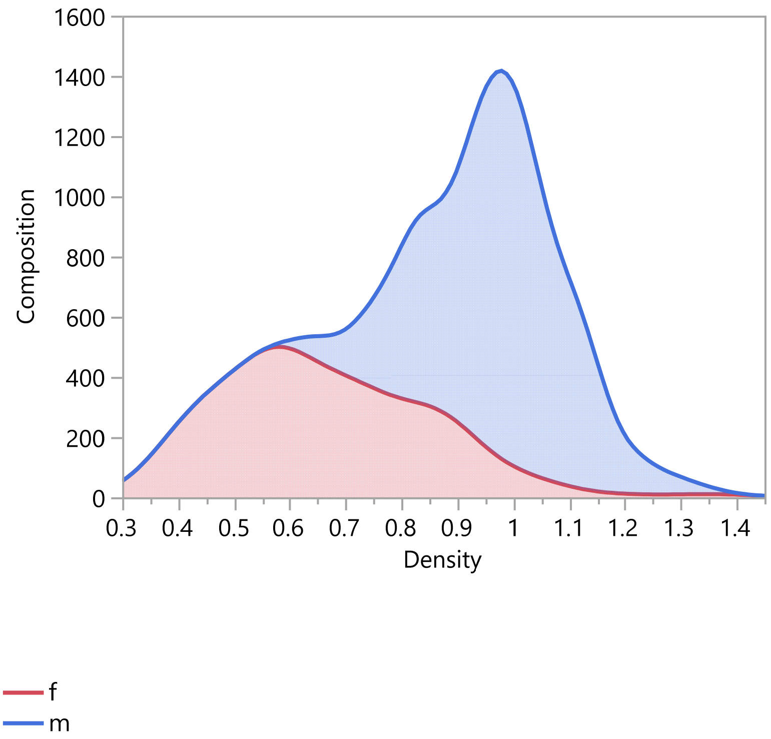

Means Comparisons

Comparisons for each pair using Student's t

Confidence Quantile

t	Alpha
1.96385	0.05

Difference Matrix

Dif=Mean[i]-Mean[j]

	m	f
m	0.00000	0.30396
f	-0.30396	0.00000

LSD Threshold Matrix

Abs(Dif)-LSD

	m	f
m	-0.02195	0.27902
f	0.27902	-0.02759

Positive values show pairs of means that are significantly different.

Connecting Letters Report

Level			Mean
m	A		0.96931993
f		B	0.66536134

Levels not connected by same letter are significantly different.

Ordered Differences Report

Level	- Level	Difference	Std Err Dif	Lower CL	Upper CL	p-Value	
m	f	0.3039586	0.0126968	0.2790239	0.3288933	<.0001*	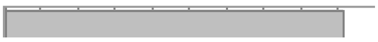

Detailed Comparisons Report

Comparing m with f

Difference	0.303959	t Ratio	23.93969
Std Err Dif	0.012697	DF	612
Upper CL Dif	0.328893	Prob > |t|	<.0001*
Lower CL Dif	0.279024	Prob > t	<.0001*

Confidence	0.95	Prob < t	1.0000

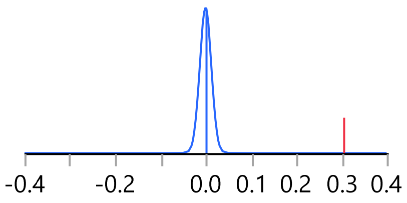

Missing Rows62

Oneway Analysis of Density By Race

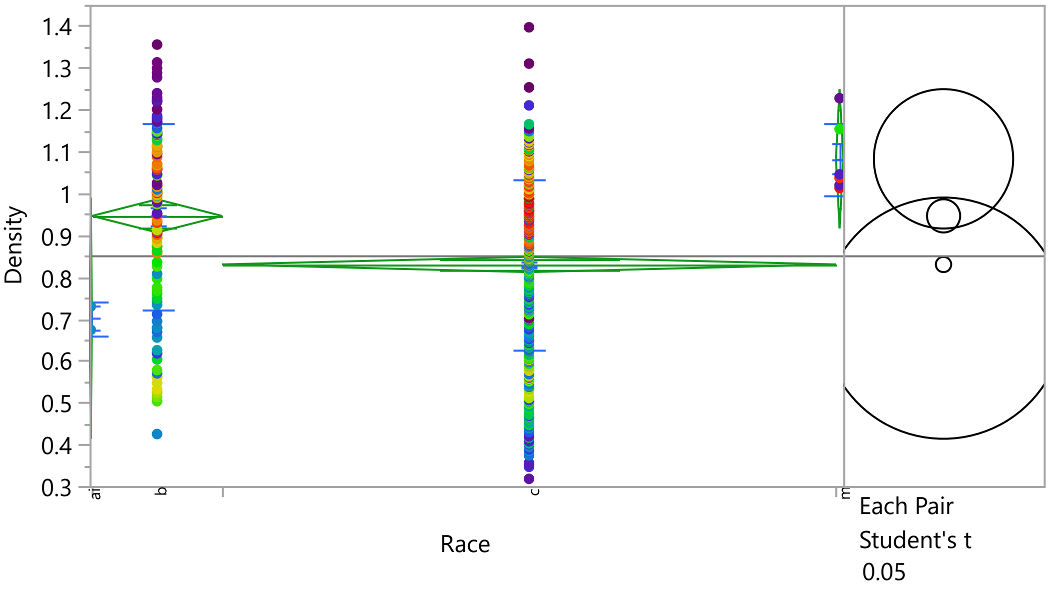

Oneway Anova

Summary of Fit

Rsquare	0.055565
Adj Rsquare	0.05092
Root Mean Square Error	0.207632
Mean of Response	0.851499
Observations (or Sum Wgts)	614

Analysis of Variance

Source	DF	Sum of Squares	Mean Square	F Ratio	Prob > F
Race	3	1.547201	0.515734	11.9629	<.0001*
Error	610	26.297743	0.043111		
C. Total	613	27.844944			

Means for Oneway Anova

Level	Mean	Std Error	Lower 95%	Upper 95%
ai	0.70124	0.14682	0.41291	0.9896
b	0.94560	0.02017	0.90600	0.9852
c	0.82938	0.00929	0.81115	0.8476
m	1.08200	0.08477	0.91553	1.2485

Std Error uses a pooled estimate of error variance

Means and Std Deviations

Level	Mean	Std Dev	Std Err Mean	Lower 95%	Upper 95%
ai	0.7012449	0.0395172	0.0279429	0.3461965	1.0562932
b	0.9456043	0.2218004	0.0215432	0.9028882	0.9883204
c	0.8293835	0.2055948	0.0091945	0.8113188	0.8474482
m	1.0820001	0.0875512	0.0357426	0.9901208	1.1738794

Means Comparisons

Comparisons for each pair using Student's t

Confidence Quantile

t	Alpha
1.96386	0.05

LSD Threshold Matrix

Abs(Dif)-LSD

	m	b	c	ai
m	-0.23542	-0.03472	0.08515	0.04782
b	-0.03472	-0.05601	0.07262	-0.04668
c	0.08515	0.07262	-0.02579	-0.16077
ai	0.04782	-0.04668	-0.16077	-0.40776

Positive values show pairs of means that are significantly different.

Levels not connected by same letter are significantly different.

Ordered Differences Report

Level	- Level	Difference	Std Err Dif	Lower CL	Upper CL	p-Value
m	ai	0.3807553	0.1695308	0.047820	0.7136902	0.0251*
m	c	0.2526166	0.0852725	0.085153	0.4200799	0.0032*
b	ai	0.2443594	0.1481966	-0.046678	0.5353969	0.0997
m	b	0.1363958	0.0871314	-0.034718	0.3075098	0.1180
c	ai	0.1281386	0.1471114	-0.160768	0.4170448	0.3841
b	c	0.1162208	0.0222020	0.072619	0.1598225	<.0001*

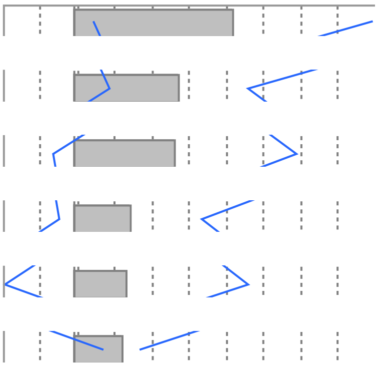

Detailed Comparisons Report

Comparing b with ai

Difference	0.24436	t Ratio	1.648887
Std Err Dif	0.14820	DF	610
Upper CL Dif	0.53540	Prob > |t|	0.0997
Lower CL Dif	-0.04668	Prob > t	0.0498*
Confidence	0.95	Prob < t	0.9502

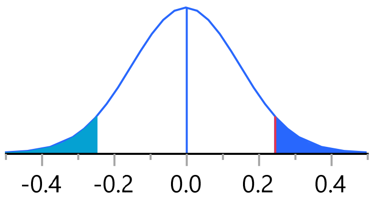

Comparing c with ai

Difference	0.12814	t Ratio	0.871032
Std Err Dif	0.14711	DF	610
Upper CL Dif	0.41704	Prob > |t|	0.3841
Lower CL Dif	-0.16077	Prob > t	0.1920
Confidence	0.95	Prob < t	0.8080

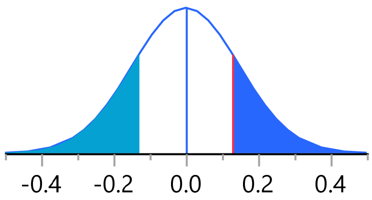

Comparing c with b

Difference	-0.11622	t Ratio	-5.23469
Std Err Dif	0.02220	DF	610
Upper CL Dif	-0.07262	Prob > |t|	<.0001*
Lower CL Dif	-0.15982	Prob > t	1.0000
Confidence	0.95	Prob < t	<.0001*

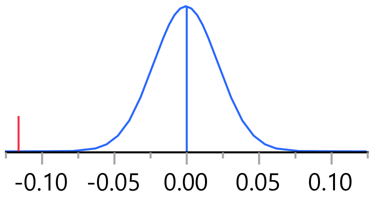

Comparing m with ai

Difference	0.380755	t Ratio	2.245935
Std Err Dif	0.169531	DF	610
Upper CL Dif	0.713690	Prob > |t|	0.0251*
Lower CL Dif	0.047820	Prob > t	0.0125*
Confidence	0.95	Prob < t	0.9875

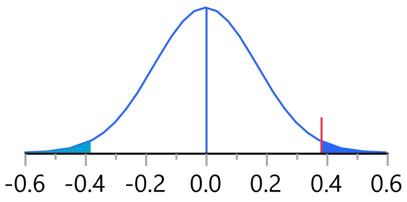

Comparing m with b

Difference	0.13640	t Ratio	1.565404
Std Err Dif	0.08713	DF	610
Upper CL Dif	0.30751	Prob > |t|	0.1180
Lower CL Dif	-0.03472	Prob > t	0.0590
Confidence	0.95	Prob < t	0.9410

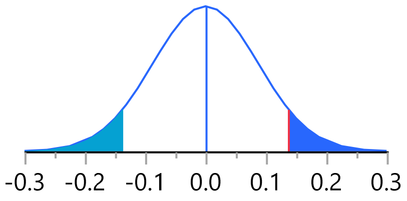

Comparing m with c

Difference	0.252617	t Ratio	2.962463
Std Err Dif	0.085272	DF	610
Upper CL Dif	0.420080	Prob > |t|	0.0032*
Lower CL Dif	0.085153	Prob > t	0.0016*
Confidence	0.95	Prob < t	0.9984

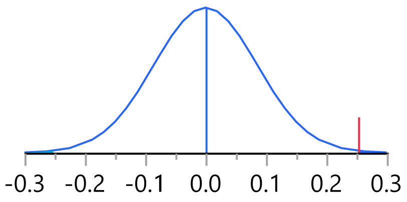

CDF Plot

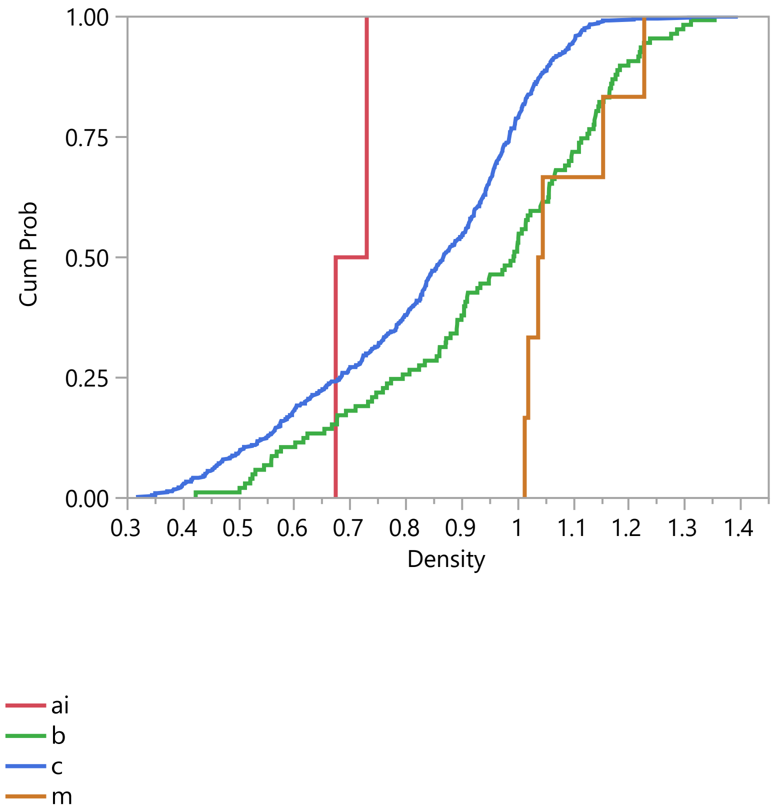

Compare Densities

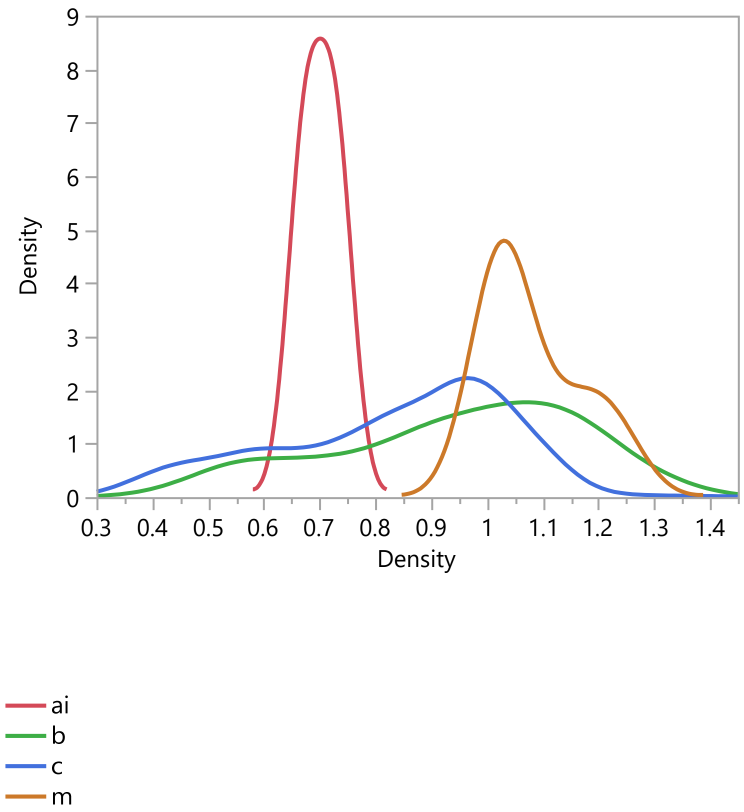

Missing Rows62

Bivariate Fit of Density By BMI

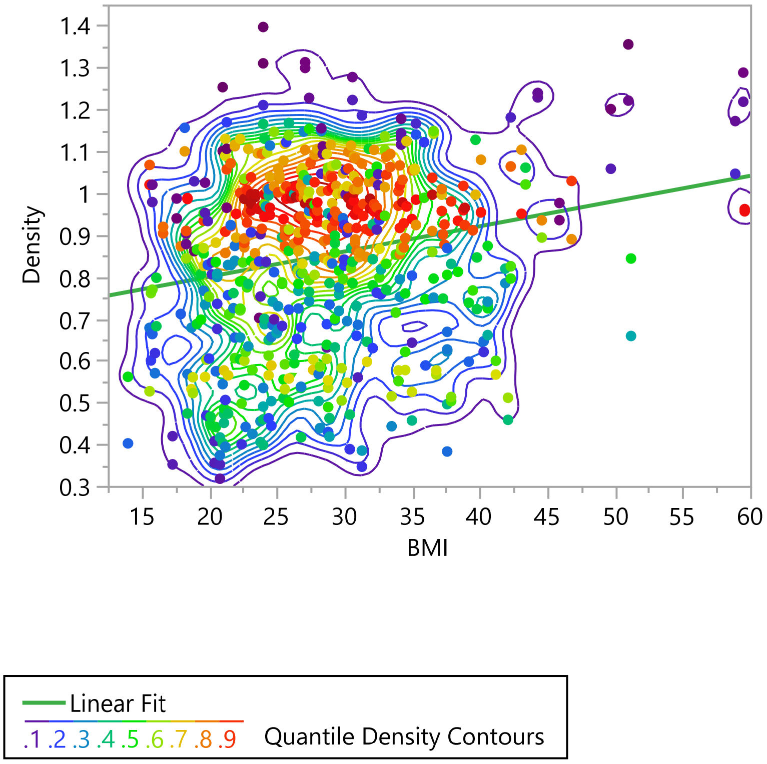

Linear Fit

Density = 0.6791666 + 0.0060304*BMI

Summary of Fit

RSquare	0.044759
RSquare Adj	0.043198
Root Mean Square Error	0.208475
Mean of Response	0.851499
Observations (or Sum Wgts)	614

Analysis of Variance

Source	DF	Sum of Squares	Mean Square	F Ratio
Model	1	1.246308	1.24631	28.6759
Error	612	26.598636	0.04346	**Prob > F**
C. Total	613	27.844944		<.0001*

Parameter Estimates

Term	Estimate	Std Error	t Ratio	Prob>|t|
Intercept	0.6791666	0.033263	20.42	<.0001*
BMI	0.0060304	0.001126	5.35	<.0001*

Diagnostics Plots

Residual by Predicted Plot

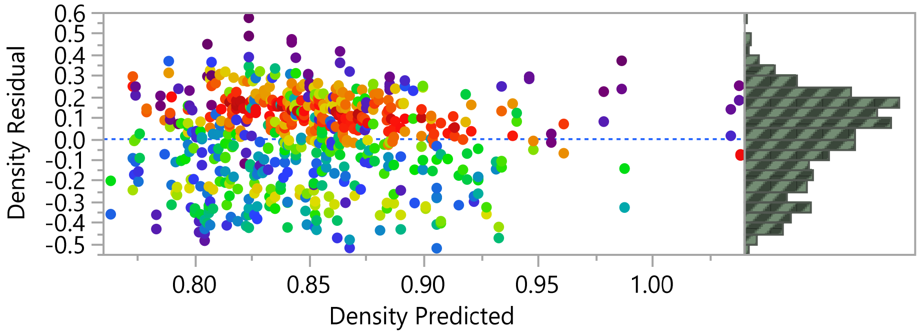

Actual by Predicted Plot

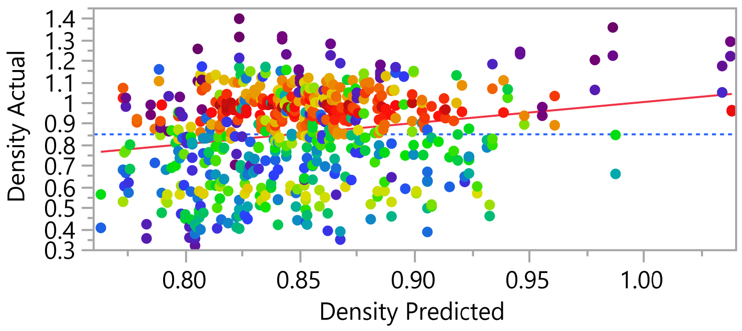

Residual by Row Plot

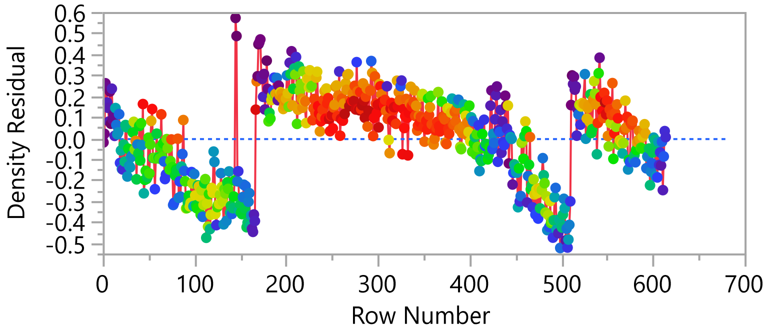

Residual by X Plot

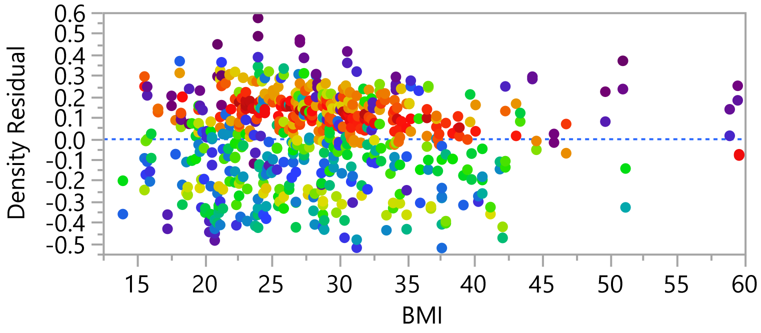

Residual Normal Quantile Plot

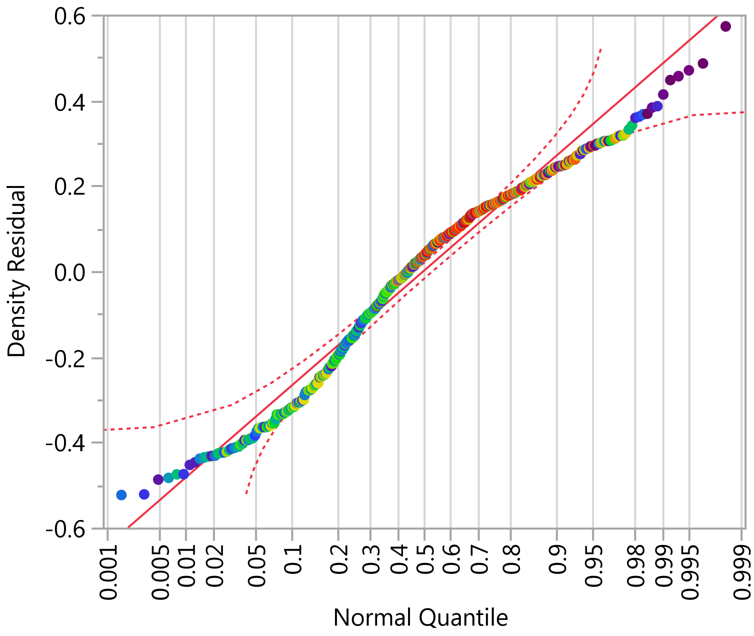

Quantile Density Contours

Variable	Kernel Std
BMI	1.282373
Density	0.036553

Bivariate Fit of Density By Weight (lbs)

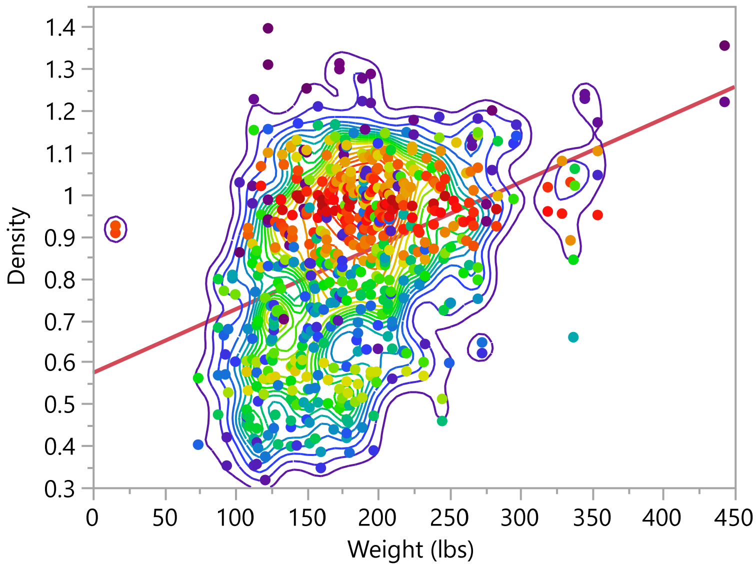

Linear Fit
.1 .2 .3 .4 .5 .6 .7 .8 .9 Quantile Density Contours

Linear Fit

Density = 0.5729131 + 0.0015183*Weight (lbs)

Summary of Fit

RSquare	0.149399
RSquare Adj	0.14801
Root Mean Square Error	0.196725
Mean of Response	0.851499
Observations (or Sum Wgts)	614

Analysis of Variance

Source	DF	Sum of Squares	Mean Square	F Ratio
Model	1	4.160019	4.16002	107.4917
Error	612	23.684925	0.03870	**Prob > F**
C. Total	613	27.844944		<.0001*

Parameter Estimates

Term	Estimate	Std Error	t Ratio	Prob>|t|
Intercept	0.5729131	0.028019	20.45	<.0001*

Term	Estimate	Std Error	t Ratio	Prob>|t|
Weight (lbs)	0.0015183	0.000146	10.37	<.0001*

Diagnostics Plots

Residual by Predicted Plot

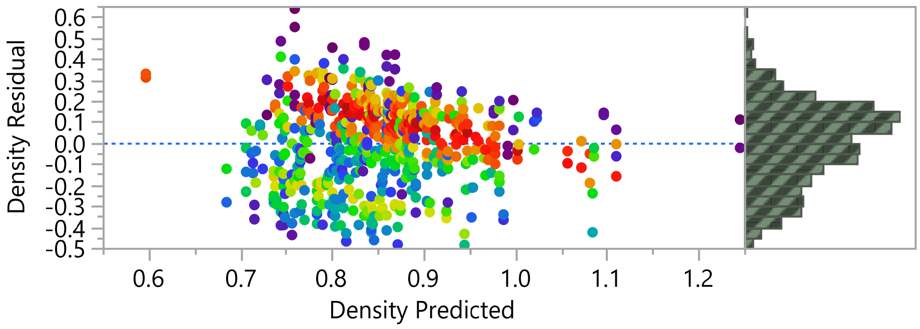

Actual by Predicted Plot

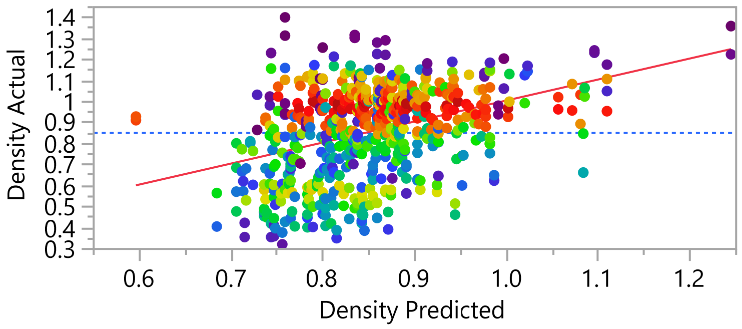

Residual by Row Plot

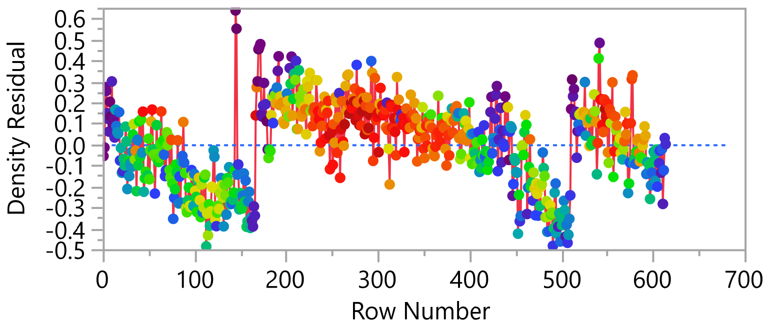

Residual by X Plot

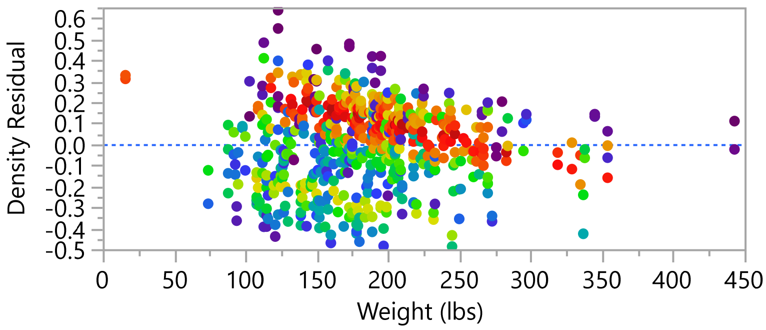

Residual Normal Quantile Plot

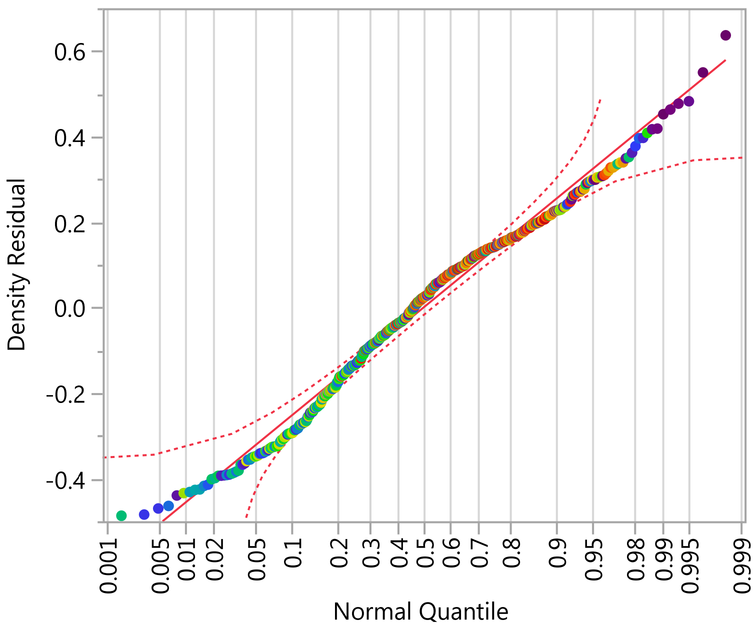

Quantile Density Contours

Variable	Kernel Std
Weight (lbs)	9.305238
Density	0.036553

Bivariate Fit of Density By Height (feet, inches)

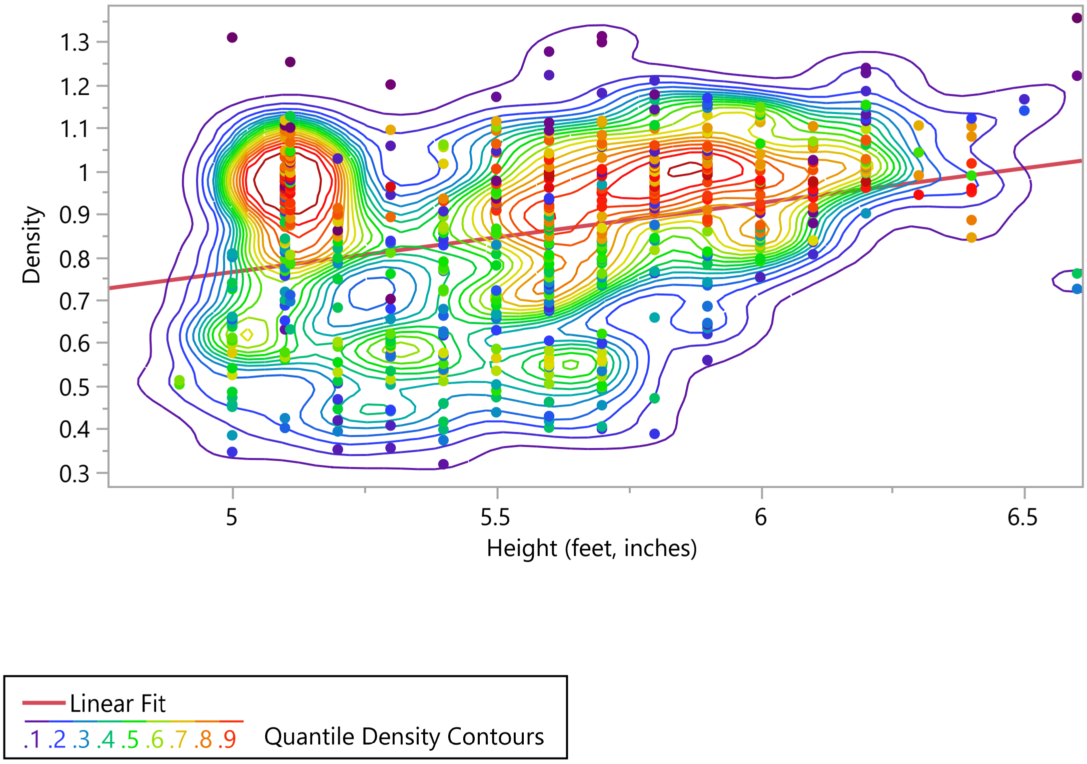

Linear Fit

Density = -0.040764 + 0.1609694*Height (feet, inches)

Summary of Fit

RSquare	0.110172
RSquare Adj	0.108718
Root Mean Square Error	0.20121
Mean of Response	0.851499
Observations (or Sum Wgts)	614

Analysis of Variance

Source	DF	Sum of Squares	Mean Square	F Ratio
Model	1	3.067720	3.06772	75.7730
Error	612	24.777224	0.04049	**Prob > F**
C. Total	613	27.844944		<.0001*

Parameter Estimates

Term	Estimate	Std Error	t Ratio	Prob>|t|
Intercept	-0.040764	0.102824	-0.40	0.6919
Height (feet, inches)	0.1609694	0.018492	8.70	<.0001*

Diagnostics Plots

Residual by Predicted Plot

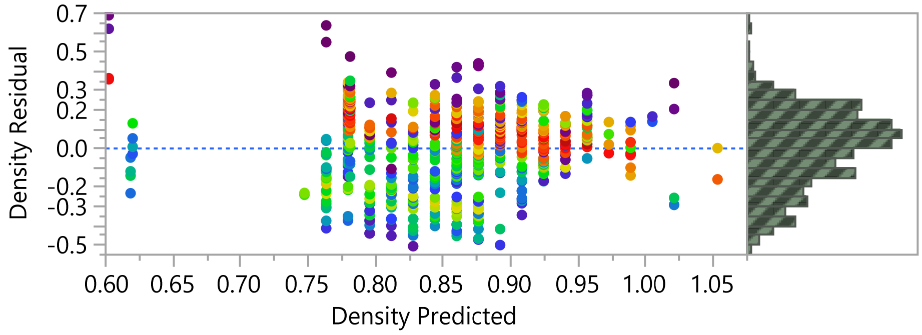

Actual by Predicted Plot

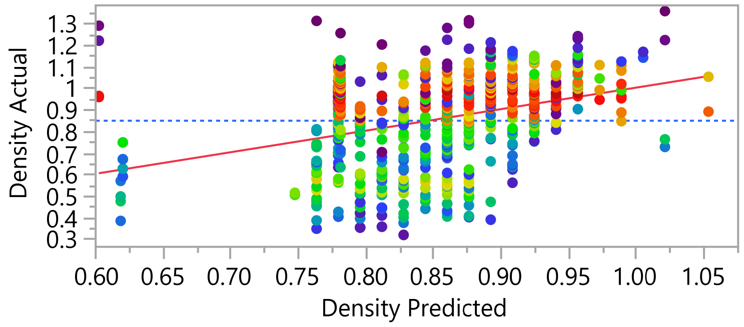

Residual by Row Plot

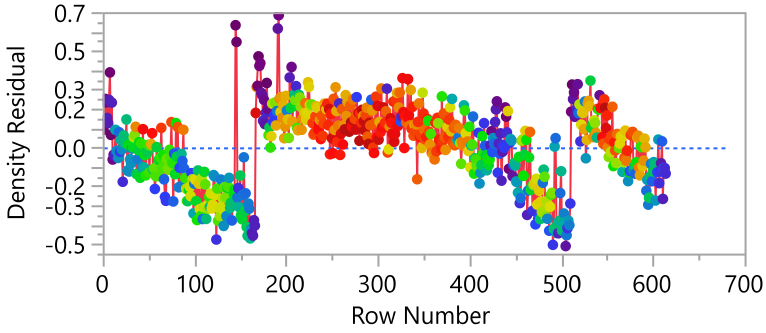

Residual by X Plot

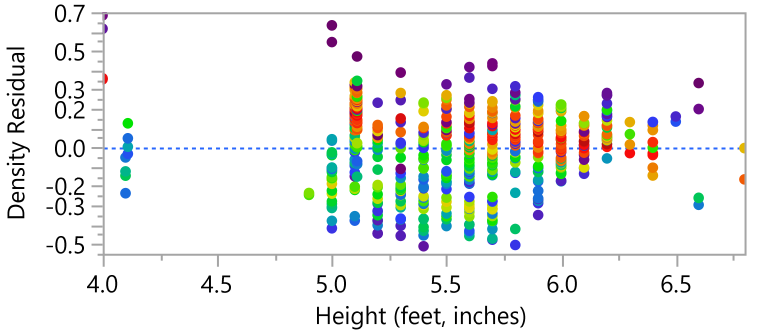

Residual Normal Quantile Plot

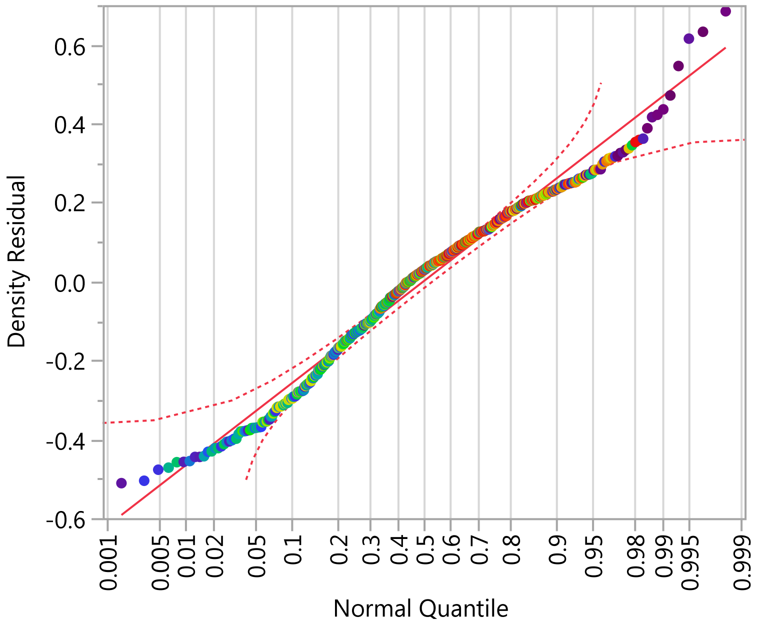

Quantile Density Contours

Variable	Kernel Std
Height (feet, inches)	0.075372
Density	0.036553

Bivariate Fit of Density By Maximum von Mises Stress(MPa)

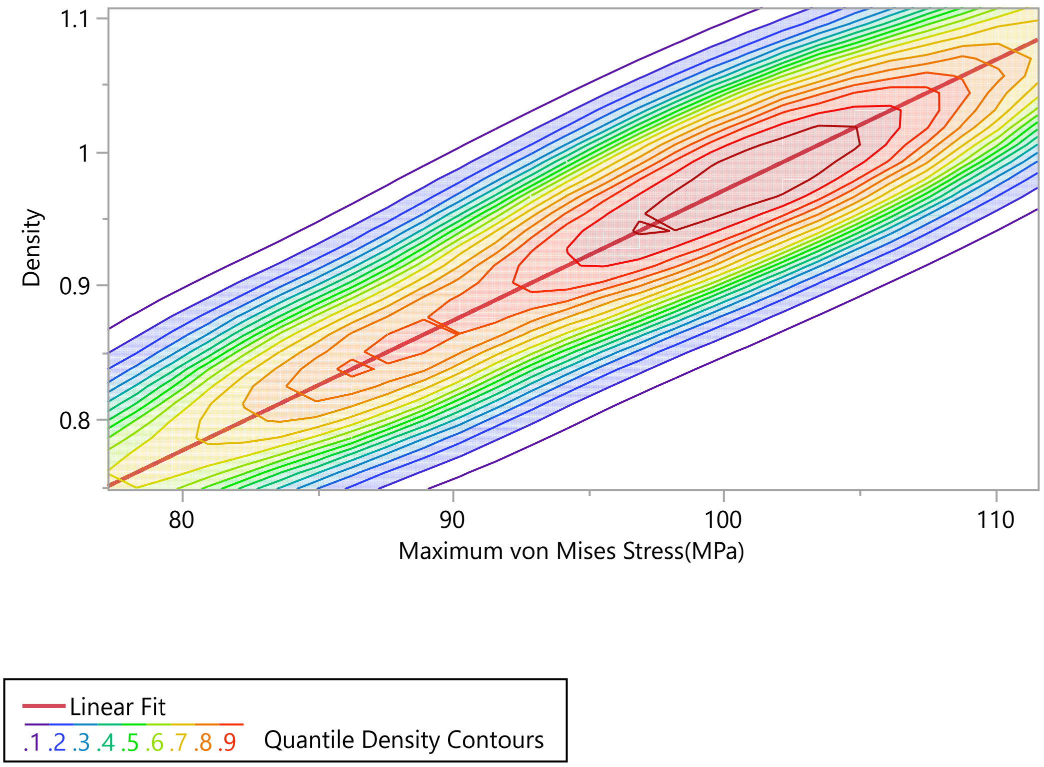

Linear Fit

Density = 4.352e-11 + 0.0097057*Maximum von Mises Stress(MPa)

Summary of Fit

RSquare	1
RSquare Adj	1

Root Mean Square Error	0
Mean of Response	0.851499
Observations (or Sum Wgts)	614

Analysis of Variance

Source	DF	Sum of Squares	Mean Square	F Ratio
Model	1	27.844944	27.8449	.
Error	612	0.000000	0.0000	**Prob > F**
C. Total	613	27.844944		.

Parameter Estimates

Term	Estimate	Std Error	t Ratio	Prob>|t|
Intercept	4.352e-11	0	.	.
Maximum von Mises Stress(MPa)	0.0097057	0	.	.

Diagnostics Plots

Residual by Predicted Plot

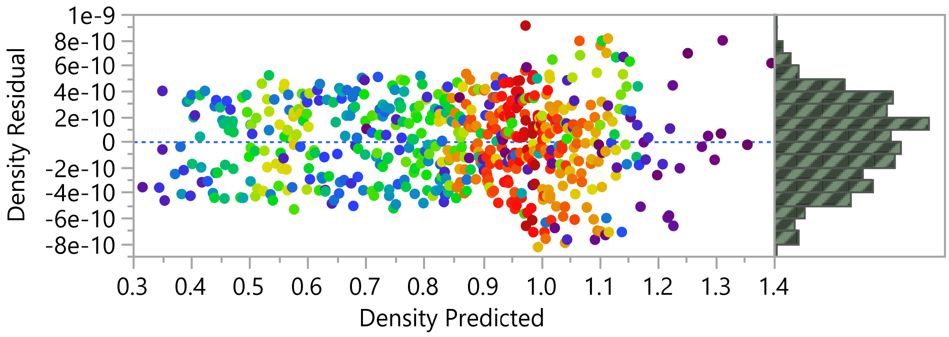

Actual by Predicted Plot

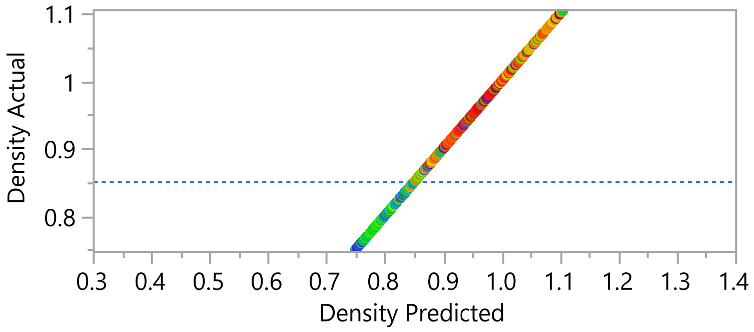

Residual by Row Plot

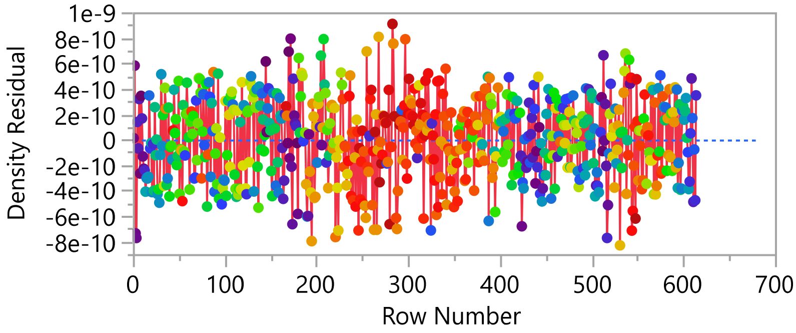

Residual by X Plot

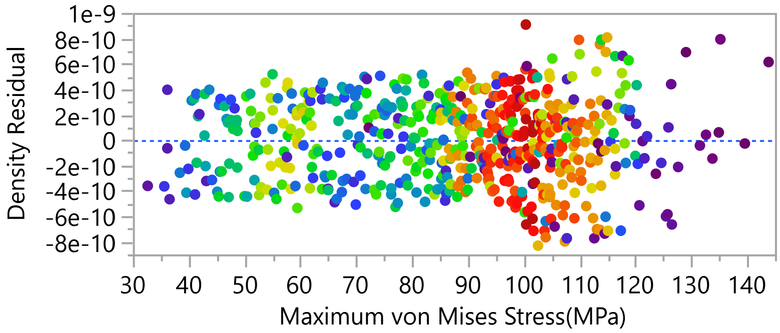

Residual Normal Quantile Plot

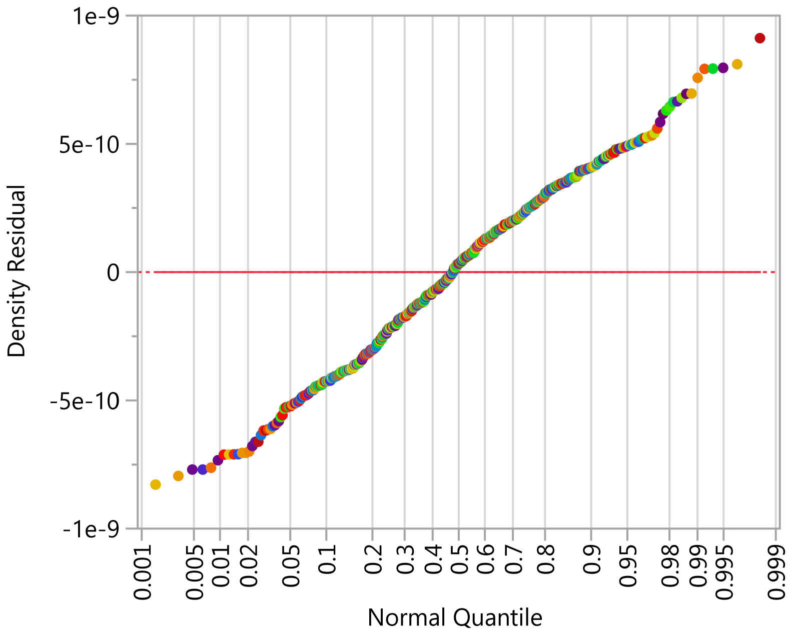

Quantile Density Contours

Variable	Kernel Std
Maximum von Mises Stress(MPa)	3.766087
Density	0.036553
